# Amelioration of Insulin Resistance by Whey Protein in a High-Fat Diet-Induced Pediatric Obesity Male Mouse Model

**DOI:** 10.3390/nu16111622

**Published:** 2024-05-25

**Authors:** Kengo Matsuda, Nobuhiko Nagano, Kimitaka Nakazaki, Daichi Katayama, Wataru Tokunaga, Koh Okuda, Shoichi Shimizu, Ryoji Aoki, Kazumasa Fuwa, Keisuke Shirai, Kazumichi Fujioka, Ichiro Morioka

**Affiliations:** 1 Department of Pediatrics and Child Health, Nihon University School of Medicine, Tokyo 173-8610, Japan; matsuda.kengo@nihon-u.ac.jp (K.M.); nakazaki.kimitaka@nihon-u.ac.jp (K.N.); daichi.katayama0509@gmail.com (D.K.); tokunaga.wataru@nihon-u.ac.jp (W.T.); kohokuda615@gmail.com (K.O.); shimizu.shoichi@nihon-u.ac.jp (S.S.); aoki.ryoji@nihon-u.ac.jp (R.A.); fuwa.kazumasa@nihon-u.ac.jp (K.F.); morioka.ichiro@nihon-u.ac.jp (I.M.); 2 Department of Pediatrics, Kobe University Graduate School of Medicine, Kobe 650-0017, Japan; ksk1024@med.kobe-u.ac.jp (K.S.); fujiokak@med.kobe-u.ac.jp (K.F.)

**Keywords:** antioxidant effect, anti-inflammatory effect, insulin resistance, metabolite analyses, peroxisome proliferator-activated receptor alpha

## Abstract

This study examined whey protein’s impact on insulin resistance in a high-fat diet-induced pediatric obesity mouse model. Pregnant mice were fed high-fat diets, and male pups continued this diet until 8 weeks old, then were split into high-fat, whey, and casein diet groups. At 12 weeks old, their body weight, fasting blood glucose (FBG), blood insulin level (IRI), homeostatic model assessment for insulin resistance (HOMA-IR), liver lipid metabolism gene expression, and liver metabolites were compared. The whey group showed significantly lower body weight than the casein group at 12 weeks old (*p* = 0.034). FBG was lower in the whey group compared to the high-fat diet group (*p* < 0.01) and casein group (*p* = 0.058); IRI and HOMA-IR were reduced in the whey group compared to the casein group (*p* = 0.02, *p* < 0.01, *p* < 0.01, respectively). The levels of peroxisome proliferator-activated receptor α and hormone-sensitive lipase were upregulated in the whey group compared to the casein group (*p* < 0.01, *p* = 0.03). Metabolomic analysis revealed that the levels of taurine and glycine, both known for their anti-inflammatory and antioxidant properties, were upregulated in the whey group in the liver tissue (*p* < 0.01, *p* < 0.01). The intake of whey protein was found to improve insulin resistance in a high-fat diet-induced pediatric obesity mouse model.

## 1. Introduction

Through epidemiological studies, Barker et al. proposed the concept that birth weight determines the risk of non-communicable disease development in adulthood (Barker theory) [1]. The developmental origins of health and disease (DOHaD) theory, recently proposed, posits that the environment, from fetal to developmental stages, plays a crucial role in establishing risk factors for chronic non-communicable diseases in adulthood and old age [2]. Additionally, infants with low birth weight are more likely to become significantly obese later in life [3].

The incidence of childhood obesity is rising in both developed and developing countries. In Japan, the percentage of overweight children peaked in 2008 but has decreased in recent years. However, this percentage remains high, with slightly less than 10% recorded among upper elementary school students [4].

Childhood obesity is recognized as a risk factor for developing hypertension, abnormalities in glucose and lipid metabolism, hyperuricemia, and nonalcoholic fatty liver disease [4]. These conditions manifest not only in adulthood but also during childhood, underscoring the need for early intervention and management. Treatment for obesity typically involves both exercise and dietary therapies [4]; however, managing exercise in infancy and early childhood is challenging. As a result, dietary interventions have been explored.

Whey protein is a milk-derived protein complex primarily consisting of lactoferrin, β-lactoglobulin, α-lactalbumin, glycomacropeptide, and immunoglobulins. Generally, casein protein, rather than whey protein, is used as feed for mice [5]. Compared to casein protein, which is also derived from milk, whey protein is absorbed more quickly by the digestive tract, resulting in a more rapid increase in blood amino acid concentrations after ingestion [6,7]. Whey protein is renowned for its multiple health benefits [8]. It may improve insulin resistance and hyperglycemia by inhibiting the release of serotonin from high-fat diets and fibroblast growth factor 21 from the liver [9]. Beta-lactoglobulin, a component of whey protein, has been shown to inhibit dipeptidyl peptidase-4 activity, thus increasing incretin levels and enhancing glucose metabolism by reducing postprandial blood glucose levels [10]. Previous research has demonstrated that administering a whey protein-enriched diet to adult males for eight weeks improved the lipid profile of the subjects, indicating enhanced lipid metabolism [11]. Whey protein is also known to exhibit anti-inflammatory effects by reducing levels of interleukin-6 and tumor necrosis factor-α [12] and possess potent antioxidant properties [13].

Oxidative stress, which increases with fat accumulation in obesity [14], notably inhibits glucose uptake in muscles [15] and reduces insulin secretion from pancreatic beta cells [16]. Obesity induces chronic inflammation and insulin resistance due to abnormal cytokine production and macrophage infiltration into adipose tissue [17]. Obesity is thus associated with chronic inflammation and oxidative stress, as previously described.

Previously, we found that administering whey protein to male mice from the embryonic period had a more pronounced effect on visceral fat reduction and insulin resistance than casein protein. Moreover, this improvement was attributed to the stimulation of β-oxidation by whey protein and its superior anti-inflammatory and antioxidative properties compared to casein protein [18]. However, few studies have examined the effects of whey protein using pediatric or young-adult obesity models. In this study, we aimed to elucidate the efficacy and mechanism of whey protein in a high-fat diet-induced pediatric obesity mouse model, where pregnant mice were fed a high-fat diet, and their offspring continued this diet during childhood.

## 2. Materials and Methods

### 2.1. Experimental Animals

All experimental plans and procedures were approved by the Animal Experimentation Committee of Nihon University Itabashi Hospital (Approval ID: AP20MED0181, Approval date: 5 June 2020). Pregnant mice from the Institute of Cancer Research (ICR) were purchased from Sankyo Laboratory Services, Inc. (Tokyo, Japan) on day 2 of gestation.

### 2.2. Rearing Conditions

Upon arrival, Slc: ICR pregnant mice were fed HFD-60, a high-fat feed with a fat content of 60% [25.6% casein, 0.36% L-cystine, 6% maltodextrin, 16% α-corn starch, 5.5% sucrose, 2% soybean oil, 33% lard, 6.6% cellulose powder, 3.5% minerals, 1.0% vitamins, 0.25% choline tartrate, and 0.18% carbonic acid; calcium: 506 kcal energy; Oriental Yeast Industry Co., Ltd. (Tokyo, Japan)]. According to previous studies, the administration of a high-fat diet with a fat-to-calorie ratio of 40–60% induces obesity, fasting hyperglycemia, hyperinsulinemia, increased insulin resistance, and decreased antioxidant and anti-inflammatory capacity in mice [19,20]. After birth, male pups were administered the same HFD-60 as their mothers until 8 weeks old. Thereafter, mice were divided into three groups: high-fat, casein, or whey groups. All mice were housed at 22 ± 2 °C under 55 ± 5% humidity and 12/12 h light/dark cycle. Male mice are known to be more susceptible to the effects of a high-fat diet than female mice [21]. As male mice were used in our previous experiments [18], male mice were used in this study.

**Figure 1:**
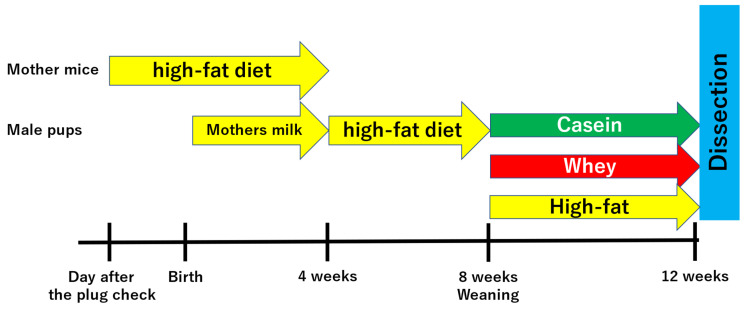
Experimental procedures.

The high-fat group was administered a high-fat diet as is. The casein group was administered AIN-93G (20% casein, 0.3% L-cystine, 39.7486% corn starch, 13.2% α-corn starch, 10.0% sucrose, 7.0% soybean oil, 5.0% cellulose powder, 3.5% minerals, 1.0% vitamins, 0.25% choline tartrate, and 0.0014% tertiary butyl hydroquinone; 359 kcal energy; Oriental Yeast Industry, Ltd., Tokyo, Japan), which is part of the standard rodent diet administered during gestation and development in mouse experiments. The whey group was administered a modified blend diet in which the casein component (AIN-93G) was replaced with whey. Male pups were fed their respective diets until 12 weeks old. Thereafter, physical and biochemical measurements were performed (Figure 1). In present study, a total of 8 mother mice and 36 male pups were used.

### 2.3. Body Weight

Pups were weighed once weekly from birth to 12 weeks old to identify any differences in weight between the three groups each week.

### 2.4. Fasting Blood Glucose Level, Serum Insulin Level, and Insulin Resistance (HOMA-IR)

Twelve-week-old adult male mice were subjected to a 12 h fast and dissected under isoflurane inhalation anesthesia (5% induction, 2% maintenance). Blood was collected from the heart via a cardiac puncture made through a mid-thoracic incision. Fasting blood glucose levels were measured using a Stat Strip XP2 (Nipro, Osaka, Japan). Serum was separated from whole blood via centrifugation at 3000 rpm for 5 min and stored at −20 °C. Serum immunoreactive insulin levels (IRI) were measured using the mouse/rat total insulin (high sensitivity) assay kit (Immunobiology Laboratories, Inc., Fujioka, Gunma, Japan). Insulin resistance was also measured using the human formula for homeostasis model evaluation of insulin resistance (HOMA-IR) [22].

### 2.5. Body Composition and Fat Weight

Body composition was measured using a bioimpedance spectroscopy device for laboratory animals (ImpediVETTM: BioResearch Center Corporation, Nagoya, Japan) [23]. To estimate fat mass (FM) and lean body mass (FFM), the difference in bioelectrical impedance for the electrical conductivity of biological tissues was measured, as adipose tissue contains less water per unit volume and is less conductive than muscle and other tissues. Fat weight was measured by removing all observable visceral adipose tissue.

### 2.6. Serum Lipoprotein Fractionation

The levels of serum lipoproteins, such as cholesterol (Cho) and triglyceride (TG), were determined via gel-permeation high-performance liquid chromatography (HPLC), according to a previously described method (LipoSEARCH^®^; Skylight Biotech, Akita, Japan) [24,25]. Cho and TG levels were estimated based on the peaks corresponding to different lipoprotein particle sizes in the HPLC elution profiles for 10 major lipoprotein classes [chylomicrons (CM), very-low-density lipoproteins (VLDL), low-density lipoproteins (LDL), high-density lipoproteins (HDL)] [25].

### 2.7. Oxidative Stress Markers

Derivatives of reactive oxidative metabolites (d-ROM) and biological antioxidant potential (BAP) were measured in the serum of 12-week-old mice using FREE Carrio Duo (WISMERLL, Tokyo, Japan). Furthermore, the oxidant to antioxidant (d-ROM/BAP) ratio was calculated as the oxidative stress index (OSI), as previously described [26].

### 2.8. Gene Expression Analysis of the Liver Tissue

The expression levels of genes related to lipid metabolism in the liver [peroxisome proliferator-activated receptor (PPAR)α, PPARγ, hormone sensitive lipase (HSL), and lipoprotein lipase (LPL)] were measured using real-time quantitative polymerase chain reaction (RT-qPCR). RNA was isolated from the frozen liver tissue of male mice (n = 5 per group), according to the protocols provided by ReliaPrep RNA Miniprep Systems (Promega Corporation, Madison, WI, USA). RNA was reverse transcribed into complementary DNA using ReverTra Ace qPCR RT Master Mix (Toyobo Co., Ltd., Osaka, Japan) on an ABI Geneamp 9700 PCR-Thermal Cycler (Applied Biosystems, Thermo Fisher Scientific Inc., Tokyo, Japan).

RT-qPCR was performed using KOD-Plus-Ver.2 polymerase mix (Toyobo Co., Ltd.) on an ABI Applied Biosystems 7300 Real-Time PCR System (Applied Biosystems, Thermo Fisher Scientific Inc.). In this study, the primers [manufactured by Takara Bio Co., Ltd. (Gunma, Japan)] used in a previous study were employed [27]. The sequences are shown in Table 1.

### 2.9. Metabolome Analysis of the Liver Tissue

Briefly, 1500 µL of 50% acetonitrile solution (*v*/*v*) (internal standard concentration: 10 µM) was added to a crushing tube containing frozen liver tissue samples (approximately 40 mg, n = 5 per group) from male mice and crushed (1500 rpm, 120 s × 3 times) using a crushing machine under cool conditions. After the crushed tissue was centrifuged (2300× *g*, 4 °C, 5 min), the supernatant was transferred to an ultrafiltration tube (Ultrafree MC PLHCC, HMT, centrifugal filter unit, 5 kDa), centrifuged (9100× *g*, 4 °C, 120 min), and subjected to ultrafiltration. The filtrate was dried and dissolved in Milli-Q water.

The solution was subjected to automatic testing using an Agilent CE system [Agilent Technologies, Inc. integrated software (Keio University, Shizuoka, Japan)]. The mass spectrum peak (range: 50–1000 *m*/*z*) area, *m*/*z*, and migration time were calculated for the detected peaks [28]. The chemical species associated with each peak were identified based on the *m*/*z* values and migration times by referencing the HMT metabolite database. The relative amounts of each metabolite were calculated by normalizing the peak areas with internal standards and sample volumes. Principal component analysis and hierarchical cluster analysis were performed as previously described [29].

### 2.10. Statistical Analysis

Data are presented as mean ± standard error. For comparisons involving three groups, the Steel–Dwass test was used. For comparisons between two groups, the Mann–Whitney U test was used when n was 6 or more in each group (e.g., comparing the experimental group (whey) to the control group (casein)); however, when n was less than 6, the Welch *t*-test was employed. JMP statistical software (ver.14.0: SAS Institute, Cary, NC, USA) was used for the analysis. *p* < 0.05 was considered to indicate statistical significance.

## 3. Results

### 3.1. Weight Change

**Figure 2:**
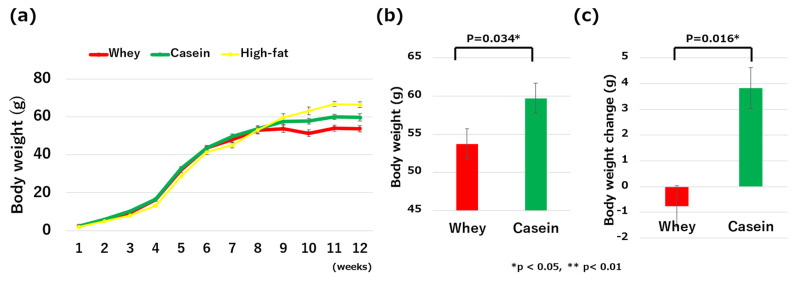

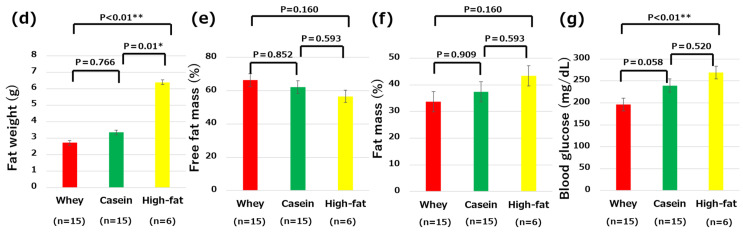
Comparisons of the clinical and metabolic parameters: (**a**) changes in body weight from birth to 12 weeks old; (**b**) body weight on the last day of high-fat-diet intake; (**c**) body weight changes from 8 to 12 weeks of age; (**d**) fat weight; (**e**) free fat mass; (**f**) fat mass; and (**g**) fasting blood glucose levels. * *p* < 0.05, ** *p* < 0.01.

Body weights did not significantly differ among all groups of mice from 5 to 9 weeks old. However, at 10 weeks of age, the mice in the whey group weighed significantly less than those in the casein group (51.42 g vs. 57.78 g, *p* = 0.042) and significantly less than those in the high-fat group (51.42 g vs. 63.23 g, *p* < 0.01). At 12 weeks, body weight was significantly less in the whey group than in the casein group (53.73 g vs. 59.74 g, *p* = 0.034) (Figure 2a,b). Weight change from 8 to 12 weeks was also significantly less in the whey group (−0.76 g vs. 3.84 g, *p* = 0.016) (Figure 2c).

### 3.2. Fat Weight and Body Composition

The fat weight did not significantly differ between the whey and casein groups of mice (2.71 g vs. 3.34 g, *p* = 0.766). However, the fat mass was significantly greater in the high-fat group compared to both the whey group (2.71 g vs. 6.38 g, *p* < 0.01) and casein group (3.34 g vs. 6.38 g, *p* = 0.01) (Figure 2d). Body composition, particularly FFM (66.34% in the whey group vs. 62.17% in the casein group vs. 53.67% in the high-fat group) and FM (33.64% in the whey group vs. 37.41% in the casein group vs. 43.36% in the high-fat group), were not significantly different among all groups (Figure 2e,f).

### 3.3. Fasting Blood Glucose

Fasting blood glucose was significantly lower in the whey group compared to the high-fat group (196.2 mg/dL vs. 268.83 mg/dL, *p* < 0.01). It also trended lower in the whey group than in the casein group (196.2 mg/dL vs. 239.73 mg/dL, *p* = 0.058). There was no significant difference in the fasting blood glucose levels of the casein and high-fat groups (239.73 mg/dL vs. 268.83 mg/dL, *p* = 0.52) (Figure 2g).

### 3.4. IRI, and HOMA-IR

**Figure 3:**
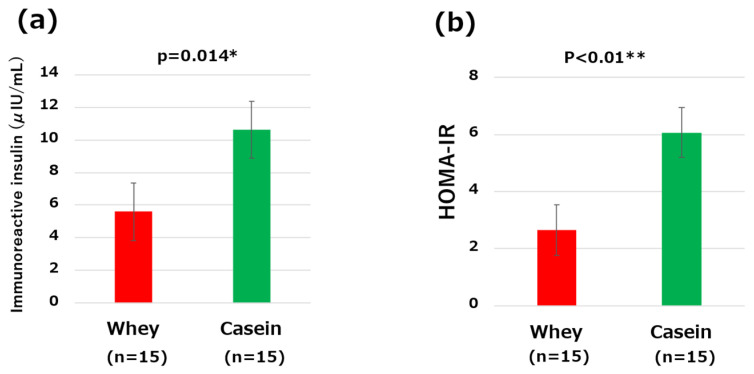
Comparisons of the clinical and metabolic parameters: (**a**) serum immunoreactive insulin levels and (**b**) homeostasis model assessment of insulin resistance (HOMA-IR) levels. * *p* < 0.05, ** *p* < 0.01.

IRI and HOMA-IR were significantly lower in the whey group than the casein group (IRI: 5.6 μIU/mL vs. 10.62 μIU/mL, *p* = 0.01; HOMA-IR: 2.65 vs. 6.06, *p* < 0.01) (Figure 3a,b).

### 3.5. Serum Lipoprotein Fractionation

Cholesterol levels did not significantly differ between the two groups of mice. However, the level of LDL-TG tended to decrease in the whey group (14.29 mg/dL vs. 17.17 mg/dL, *p* = 0.09) (Table 2).

### 3.6. Oxidative Stress, Biological Antioxidant Capacity, and Oxidative Stress Index

**Figure 4:**
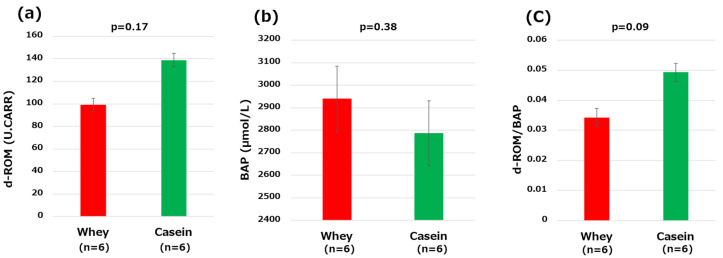
Oxidative stress markers: (**a**) derivatives of reactive oxidative metabolites (d-ROMs); (**b**) biological antioxidant potential (BAP); and (**c**) oxidative stress index (OSI, d-ROM/BAP).

The d-ROMs (99.16 vs. 138.83, *p* = 0.17) and BAP (2939.83 μmol/L vs. 2786.33 μmol/L, *p* = 0.38) did not significantly differ between the two groups of mice. The OSI tended to be lower in the whey group compared to the casein group (0.03 vs. 0.05 *p* = 0.09) (Figure 4a–c).

### 3.7. Analysis of Genes Related to Lipid Metabolism in the Liver

**Figure 5:**
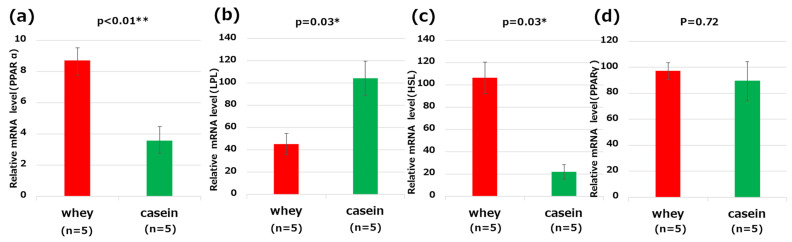
Genes related to lipid metabolism in the liver: (**a**) peroxisome proliferator-activated receptor (PPAR)α; (**b**) lipoprotein lipase (LPL); (**c**) hormone sensitive lipase (HSL); and (**d**) peroxisome proliferator-activated receptor (PPAR)γ. * *p* < 0.05, ** *p* < 0.01.

Based on RT-qPCR, mice in the whey group had significantly higher expression levels of PPARα and HSL in the liver than those in the casein group (PPARα: *p* < 0.01, HSL: *p* = 0.03). The expression level of LPL was significantly lower in the whey group than in the casein group (*p* = 0.03). PPARγ was not found to significantly differ between the two groups of mice (*p* = 0.72) (Figure 5a–d).

### 3.8. Metabolome Analysis of the Liver

**Figure 6:**
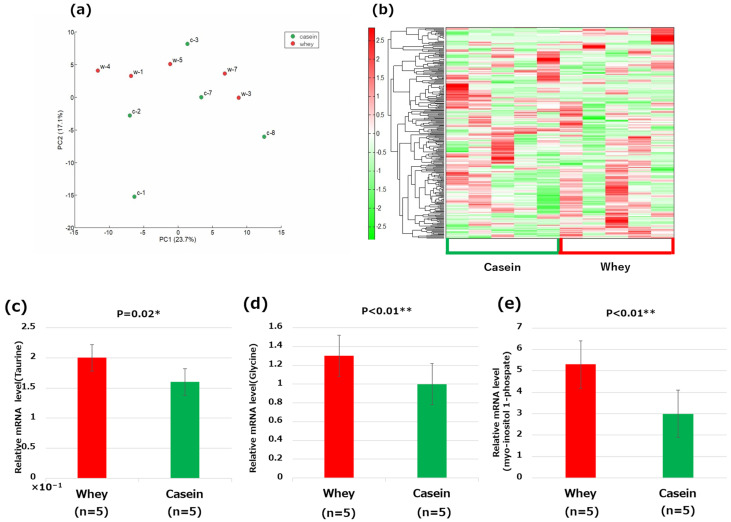
Metabolome analysis of the liver: (**a**) principal component (PC) analysis; (**b**) heat map of the hierarchical cluster analysis; (**c**) taurine; (**d**) glycine; and (**e**) myo-inositol 1-phosphate. * *p* < 0.05, ** *p* < 0.01.

Principal component analysis or hierarchical clustering heat mapping did not reveal any clear differences between the groups; however, individual significant differences were found (Figure 6a,b. Supplementary Tables S1–S3). In particular, the levels of taurine, glycine, and myo-inositol 1-phosphate were significantly elevated in the whey group compared to those in the casein group (taurine: *p* = 0.02, glycine: *p* < 0.01, and myoinositol 1-phosphate: *p* < 0.01) (Figure 6c–e).

## 4. Discussion

In the present study, conducted on a high-fat diet-induced pediatric obesity mouse model, it was found that intake of whey protein resulted in the suppression of body weight gain, a lower fat mass, and improved fasting blood glucose levels than the casein group when compared to the high-fat group. Furthermore, whey protein intake was observed to improve insulin resistance compared to casein protein. These results suggest that the improvements in insulin resistance associated with whey protein are likely due to enhanced β-oxidation and the promotion of anti-inflammatory and antioxidant effects compared to casein protein.

### 4.1. Lipid Metabolism-Related Genes

**Figure 7:**
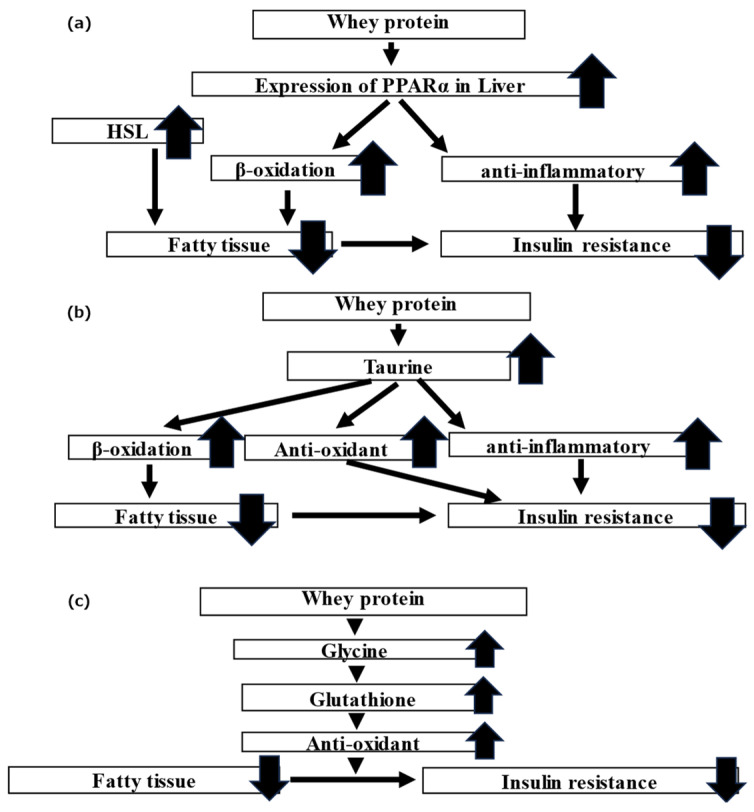
Schematic of the theory for the mechanism by which whey protein ameliorates the metabolism of lipid and glucose: (**a**) PPARα, HSL; (**b**) taurine; and (**c**) glycine.

Mice fed whey protein exhibited improved fasting blood glucose levels and insulin resistance compared to those fed casein protein. In terms of body composition and visceral fat mass, the average lean body mass of the mice in the whey group was higher while the average visceral fat mass was lower than those of the mice in the casein group. Furthermore, the mice in the whey group had significantly higher expressions of *PPARα* and *HSL* in the liver than those in the casein group. Previously, Sasaki et al. [27] revealed that the intake of whey protein affected the expression of *PPARα*, *PPARγ*, and *SREBP1c* in the gastrocnemius, liver, and epididymis adipose tissue of mice, as well as their downstream enzymes, HSL, LPL, ACCα, and FAS. PPARα is known to promote β-oxidation of fatty acids in intracellular mitochondria to stimulate lipid metabolism [30] and has anti-inflammatory effects [31]. Inflammatory chemokines and cytokines are known to affect insulin resistance [32,33]. HSL breaks down adipocyte TG into free fatty acids and glycerol [34] and may promote β-oxidation [35]. In the present study, we inferred that whey protein intake enhances the reduction of visceral fat and improves insulin resistance by increasing the expression of *PPARα* and *HSL*, promoting β-oxidation of fatty acids in the liver, and leveraging the anti-inflammatory effects of PPARα itself (Figure 7a).

### 4.2. Involvement of Taurine

Metabolomic analysis revealed a significantly higher level of taurine in the whey group relative to the casein group. Taurine is a type of amino acid obtained via diet and is known to be produced from cysteine in the liver and fat cells in vivo [36]. According to a previous study, taurine is more abundant in breast milk than cow’s milk, and powdered milk with more whey protein contains more taurine than powdered milk with the same ratio of casein protein and whey protein as cow’s milk [37]. As whey protein is rich in essential amino acids, including cysteine [38], the elevated level of taurine may occur through endogenous and exogenous paths.

Taurine improves lipid metabolism by promoting bile acid synthesis from Cho and β-oxidation of the fatty acids in the mitochondria in mice administered a high-fat diet [39,40]. Taurine is also reported to exhibit antioxidant effects and inhibit fat oxidation in the liver by improving mitochondrial function [41]. Additionally, taurine exhibits anti-inflammatory effects in adipose tissue [42]. Overall, whey protein intake may elevate taurine endogenously and exogenously and may be involved in the elevation in β-oxidation and the anti-oxidative anti-inflammatory effects (Figure 7b), ultimately leading to improved lipid and glucose metabolism.

### 4.3. Glycine and Myo-Inositol 1-Phosphate

Previously, we speculated that whey protein improves insulin resistance by increasing glutathione in adipocytes and exhibiting an antioxidant effect [18]. Glutathione is composed of glycine, cysteine, and glutamic acid. Previous research has shown that administering glycine and cysteine to diabetic patients increases glutathione levels and improves oxidative stress [43] and that administration of glycine to model mice with increased insulin resistance and oxidative stress increased glutathione and improved oxidative stress, glucose metabolism, and visceral fat mass [44]. In the present study, glycine levels were elevated in the whey group. We inferred that the administration of whey protein elevated glycine levels, which subsequently raised glutathione levels. This enhancement in glutathione likely contributed to its antioxidant effects, resulting in improved insulin resistance (Figure 7c).

In a previous study, myo-inositol 1-phosphate levels were found to be elevated by whey protein administration [18]. Similar results were obtained in a high-fat diet-induced childhood obesity mouse model. As myo-inositol is known to improve insulin resistance [45] and myo-inositol 1-phosphate belongs to the myo-inositol metabolic pathway [46], myo-inositol 1-phosphate may also play a role in improving glucose metabolism.

### 4.4. Limitations

In this study, insulin resistance was found to significantly differ between the whey and casein groups; however, no clear differences were found in the blood lipid profile, visceral fat mass, or OSI.

This result may be attributed to the use of a high-fat diet-induced pediatric obesity mouse model, which exhibits elevated fat mass, abnormal lipid metabolism, and heightened oxidative stress, in contrast to mice fed a standard diet [19,20]. In addition, the whey–casein diet was administered for a shorter period (4 weeks) in this study than other studies [9]; therefore, the difference may not be significant.

The present study measured only serum insulin levels and HOMA-IR for assessing insulin resistance, which might not be completely accurate. Future research could more precisely evaluate insulin resistance by employing the glucose clamp technique and 2-deoxyglucose glucose uptake.

The expression level of *PPARα* elevated while that of *LPL* reduced in the liver of mice in the whey group compared to those of mice in the casein group. According to a previous study, LPL is upregulated in fat and muscle and downregulated in the liver of mice administered a high-fat diet [47]. In this study, metabolomic analysis was only performed on the liver; therefore, other organs must assessed to determine LPL levels. In addition, this study evaluated only the gene expression levels of lipid metabolism-related genes. In future research, we will also assess the protein expression of these genes using Western blotting and other techniques.

### 4.5. Future Prospects

Fetal administration of whey protein [18] and administration of whey protein to mice models of high-fat diet-induced childhood obesity led to improvements in fasting blood glucose, insulin resistance, visceral fat loss, and blood lipid profiles. This study suggests that whey protein promotes β-oxidation and enhances anti-inflammatory and antioxidant effects. Low birthweight infants are at increased risk of developing type 2 diabetes in adulthood due to the accumulation of visceral fat and insufficient muscle mass [48]. In addition, oxidative stress has been found to be higher in low-birth-weight infants [49]. Whey protein may have a preventive effect on the development of type 2 diabetes in infants with a low birth weight. Feeding usually transitions from breast milk or artificial baby milk to cow’s milk. Mature milk and powdered milk have a whey and casein protein ratio of 60% to 40%, whereas cow’s milk has a reduced whey protein content of 20% and 80% casein protein [50]. The addition of whey protein during this transition period may reduce the risk of diabetes development in infants with low birth weight. However, although some reports suggest that a high-protein diet has protective effects against diabetes and obesity in adults [51], other studies indicate that a high-protein diet in early infancy may induce an early adiposity rebound [52]. Therefore, merely adding whey protein to regular nutrition could increase protein intake and potentially be counterproductive concerning future risks of diabetes, obesity, and other diseases. We plan to conduct whey protein intervention experiments using the low-birth-weight, non-obese hyperglycemic mouse model developed by Katayama et al. [53] to assess the potential of whey protein in enhancing glycolipid metabolism. In our previous studies, we administered only whey protein or casein protein. However, both breast milk and powdered milk contain a mixture of whey and casein proteins. For future clinical applications, it is necessary to conduct studies using feed that includes a mixture of whey and casein proteins in varying ratios.

## 5. Conclusions

Compared to casein protein, whey protein has been shown to improve insulin resistance in a high-fat diet-induced pediatric obesity mouse model. This improvement in insulin resistance is believed to result from enhanced beta oxidation, which is facilitated by elevated expressions of *HSL* and *PPARα*, as well as the augmented anti-inflammatory and antioxidant effects due to elevated levels of taurine and glycine.

## Figures and Tables

**Table 1:** List of DNA primer sets for quantitative RT-qPCR.

Gene	Direction	Sequence	Start Position	Size
**PPARα**	Forward	CTCAGGGTACCACTACGGAGTTCAC	1579	153
	Reverse	TGAATCTTGCAGCTCCGATCAC	1731	
**PPARγ**	Forward	GGAGCCTAAGTTTGAGTTTGCTGTG	1579	153
	Reverse	TGCAGCAGGTTGTCTTGGATG	1731	
**HSL**	Forward	TCCTGGAACTAAGTGGACGCAAG	2093	93
	Reverse	CAGACACACTCCTGCGCATAGAC	2185	
**LPL**	Forward	AGAGGCTATAGCTGGGAGCAGAAAC	3440	137
	Reverse	GCAAGGGCTAACATTCCAGCA	3616	

**Table 2:** Serum lipoprotein levels.

	Casein (n = 10)	Whey (n = 10)	*p*-Value
**Total-Cho**	159.29 (117.69–212.91)	139.2 (91.83–182.78)	0.12
**CM-Cho**	0.61 (0.31–0.85)	0.69 (0.3–1.23)	0.68
**VLDL-Cho**	11.94 (5.71–19.12)	13.04 (6.03–19.56)	0.52
**LDL-Cho**	26.18 (14.66–33.65)	19.12 (8.96–27.14)	0.16
**HDL-Cho**	120.55 (71.51–173.39)	106.33 (70.46–127.63)	0.21
**Total-TG**	95.58 (42.82–162.86)	91.37 (42.76–146.48)	0.79
**CM-TG**	4.99 (1.09–9.45)	4.69 (1–11.66)	0.85
**VLDL-TG**	69.21 (26.22–131.14)	68.64 (25.09–115.98)	0.91
**LDL-TG**	17.17 (11.72–22.78)	14.29 (9.64–24.01)	0.09
**HDL-TG**	4.21 (2.03–6.39)	3.749 (2.36–4.49)	0.24

Cho: cholesterol; CM: chylomicron; HDL: high-density lipoprotein; LDL: low-density lipoprotein; TG: triglyceride; and VLDL: very-low-density lipoprotein.

## Data Availability

The data that support the findings of this study are available from the corresponding author upon reasonable request.

## Supplementary Materials

The following supporting information can be downloaded at https://www.mdpi.com/article/10.3390/nu16111622/s1, Table S1: principal component score; Table S2: metabolites and principal component score; and Table S3: Results of comparative analysis.
