# Supplementary material for: Amelioration of Insulin Resistance by Whey Protein in a High-Fat Diet-Induced Pediatric Obesity Male Mouse Model
Source: Nutrients. 2024 May 25;16(11):1622. doi: 10.3390/nu16111622 (PMC11174045; doi:10.3390/nu16111622)
Supplement: Supplementary file 1 [file nutrients-16-01622-s001.zip › Supplementary Table S1.pdf]

Supplementary Table S1. Principal component score

	Contribution rate (%)	Whey					Casein				
		Whey1	Whey3	Whey4	Whey5	Whey7	Casein1	Casein2	Casein3	Casein7	Casein8
PC1	23.62	-6.83	8.83	-11.65	-1.14	6.80	-6.33	-7.00	1.38	3.39	12.54
PC2	17.06	3.26	-0.08	4.07	5.08	3.63	-15.25	-2.80	8.16	-0.01	-6.06
PC3	14.25	-3.91	-7.27	-5.12	4.97	-8.39	2.11	1.30	11.56	1.38	3.37
PC4	9.94	5.04	-10.60	-1.47	-1.54	5.72	-2.73	2.82	-2.33	-1.33	6.42
PC5	9.34	2.67	0.06	-9.17	0.88	6.57	4.32	1.10	2.79	-1.55	-7.69
PC6	8.73	3.66	-0.10	-2.87	-5.95	-3.25	-2.53	2.51	-0.40	11.15	-2.23
PC7	6.91	-7.79	-2.62	4.00	-5.49	5.39	1.27	1.27	3.65	2.00	-1.70
PC8	5.47	-4.10	1.30	-1.75	3.15	-0.14	-3.30	8.92	-2.97	-0.45	-0.65
PC9	4.69	2.43	3.07	-0.37	-6.04	-1.70	-1.13	3.45	3.95	-5.17	1.51

PC,principal component
