# Supplementary material for: Amelioration of Insulin Resistance by Whey Protein in a High-Fat Diet-Induced Pediatric Obesity Male Mouse Model
Source: Nutrients. 2024 May 25;16(11):1622. doi: 10.3390/nu16111622 (PMC11174045; doi:10.3390/nu16111622)
Supplement: Supplementary file 1 [file nutrients-16-01622-s001.zip › Supplementary Table S2.pdf]

Supplementary Table S2. Metabolites and principal component score

ID	Compound name	PubChem CID	HMDB ID	*m/z*	MT/RT	PC1	PC2	PC3	PC4	PC5	PC6	PC7	PC8	PC9
A_0003	Pyruvic acid	1060	HMDB0000243	87.009	10.46	2.0E-01	2.1E-01	1.2E-01	5.7E-01	3.8E-01	2.5E-01	-3.3E-01	5.1E-01	-4.0E-02
A_0004	Lactic acid	612	HMDB0000190,H	89.024	9.01	-3.3E-01	-5.8E-01	8.8E-03	6.2E-01	3.3E-01	1.6E-01	1.8E-01	-1.7E-02	2.0E-02
A_0005	3-Hydroxybutyric acid	441	HMDB0000011,H	103.040	8.09	-1.7E-01	3.4E-01	-2.3E-01	-4.3E-01	-6.3E-01	-1.1E-01	1.6E-01	-2.7E-01	3.2E-01
A_0006	2-Hydroxybutyric acid	440864	HMDB0000008	103.040	8.28	-4.8E-01	5.3E-01	1.8E-01	-1.1E-01	3.2E-01	4.2E-02	-4.1E-01	-4.1E-01	-3.2E-02
A_0007	2-Hydroxyisobutyric acid	11671	HMDB0000729	103.040	8.36	-1.1E-01	-6.3E-03	1.6E-01	1.6E-01	-6.5E-01	3.5E-01	1.1E-01	4.6E-01	-4.1E-01
A_0008	Fumaric acid	444972	HMDB0000134	115.004	17.18	-7.6E-01	-4.6E-01	-3.8E-02	1.3E-01	2.5E-01	-3.4E-01	4.2E-03	1.4E-01	3.7E-02
A_0010	*N*-Acetylglycine	10972	HMDB0000532	116.035	8.08	2.9E-01	2.6E-01	2.3E-02	-3.5E-01	-2.5E-01	-4.1E-01	-4.1E-01	-5.4E-01	1.8E-01
A_0011	Succinic acid	1110	HMDB0000254	117.019	15.22	1.9E-01	4.9E-01	3.9E-01	5.4E-01	8.2E-02	4.7E-01	2.1E-01	-6.8E-02	1.4E-02
A_0012	2-Hydroxyvaleric acid<br>2-Hydroxyisovaleric acid	98009<br>99823	HMDB0001863<br>HMDB0000407	117.056	7.88	-2.3E-01	1.9E-01	4.3E-01	-1.4E-01	3.3E-01	-5.5E-01	-1.9E-01	2.2E-01	-4.6E-01
A_0013	β-Hydroxyisovaleric acid	69362	HMDB0000754	117.056	7.68	-5.4E-01	1.4E-01	-4.6E-02	-3.3E-01	-1.2E-01	-1.2E-01	3.4E-02	-6.2E-01	4.1E-01
A_0014	Isethionic acid	7866	HMDB0003903	124.991	9.56	6.3E-02	-6.5E-01	-3.4E-01	-2.9E-02	-1.9E-01	-1.2E-01	-5.1E-01	3.7E-01	-4.3E-02
A_0015	5-Oxoproline	7405	HMDB0000267	128.035	8.03	-7.8E-01	4.6E-01	1.1E-01	-2.5E-01	1.3E-01	2.8E-01	-1.8E-02	4.2E-02	-8.9E-02
A_0016	Propionylglycine	98681	HMDB0000783	130.051	7.66	-4.2E-01	4.1E-01	-3.3E-01	-3.0E-01	-3.9E-01	-3.2E-01	-4.0E-01	-7.0E-02	-1.7E-01
A_0017	*N*-Acetylalanine<br>*N*-Acetyl-β-alanine	88064<br>76406	HMDB0000766	130.051	7.55	-7.5E-01	-4.2E-01	-3.0E-01	2.2E-01	1.3E-01	-1.0E-01	2.4E-01	1.0E-01	1.8E-01
A_0018	3-Ureidopropionic acid	111	HMDB0000026	131.046	7.83	-9.1E-02	-2.9E-02	6.6E-01	-2.4E-01	3.4E-01	-1.2E-01	3.3E-01	-4.1E-01	3.0E-01
A_0019	Iminodiacetic acid	8897	HMDB0011753	132.030	7.84	9.4E-02	7.5E-01	5.5E-01	1.6E-01	2.0E-01	-1.2E-01	1.3E-02	6.4E-02	1.8E-01
A_0020	Malic acid	525	HMDB0000156,H	133.014	15.44	-7.8E-01	-4.0E-01	-1.0E-01	8.4E-02	2.7E-01	-3.6E-01	3.8E-02	4.8E-02	7.6E-02
A_0021	Threonic acid	5460407	HMDB0000943	135.030	7.86	-2.4E-01	-2.7E-01	4.9E-02	5.9E-01	-3.1E-01	-6.5E-02	-4.9E-01	-4.2E-01	2.8E-02
A_0022	*p*-Toluic acid<br>*o*-Toluic acid<br>*m*-Toluic acid	7470<br>8373<br>7418		135.045	7.79	-7.4E-01	1.3E-01	1.8E-02	-5.6E-01	-1.7E-01	1.8E-01	-2.0E-01	-8.6E-02	-1.2E-01
A_0023	Ethanolamine phosphate	1015	HMDB0000224	140.012	6.88	-4.2E-01	6.2E-01	-4.6E-02	3.3E-01	2.7E-01	-2.3E-01	-1.5E-01	-3.1E-01	2.8E-01
A_0024	*N*-Butyrylglycine<br>Isobutyrylglycine	88412<br>10855600	HMDB0000808<br>HMDB0000730	144.067	7.35	-3.6E-01	-8.1E-01	-1.2E-01	-3.8E-02	-3.8E-01	-5.8E-02	1.8E-01	-5.2E-02	1.4E-01
A_0025	2-Oxoglutaric acid	51	HMDB0000208	145.015	15.40	-3.8E-01	1.4E-01	-7.5E-01	2.1E-02	3.1E-01	-3.5E-01	-1.9E-01	1.4E-01	9.7E-03
A_0026	2-Hydroxyglutaric acid	43	HMDB0000606,H	147.030	13.15	5.6E-02	-1.5E-01	-2.1E-01	1.8E-01	-8.4E-02	-2.8E-01	7.4E-01	4.4E-01	-2.5E-01
A_0027	Tartaric acid	444305	HMDB0000956	149.010	15.84	-5.3E-01	-6.3E-01	-3.1E-02	3.1E-01	1.3E-01	5.0E-02	-3.1E-01	-1.6E-01	3.0E-01
A_0028	3-Phenylpropionic acid	107	HMDB0000764	149.060	7.51	-2.0E-01	9.8E-02	-4.2E-01	1.5E-01	6.9E-02	-5.3E-01	-5.8E-01	3.3E-01	-1.4E-01
A_0029	Cysteinesulfinic acid	1549098	HMDB0000996	152.002	8.06	2.3E-02	3.7E-01	3.7E-01	1.6E-01	4.3E-01	-3.6E-02	5.2E-01	2.2E-01	4.4E-01
A_0030	Pelargonic acid	8158	HMDB0000847	157.123	7.00	3.5E-01	6.9E-01	-1.2E-02	4.1E-01	1.2E-01	-5.4E-02	-1.7E-01	-2.3E-01	-3.6E-01
A_0031	Isovalerylglycine<br>2-Methylbutyrylglycine	546304<br>193872	HMDB0000678<br>HMDB0000339	158.082	7.13	-7.4E-01	-4.3E-01	-1.4E-01	2.4E-01	-3.4E-01	2.0E-01	1.2E-01	-5.8E-02	1.1E-01
A_0032	*N*-Acetylthreonine	152204	HMDB0062557	160.062	7.19	-5.3E-01	2.5E-01	5.8E-01	2.7E-01	-1.6E-01	2.4E-01	-3.9E-01	-1.5E-01	-2.1E-03
A_0033	Terephthalic acid	7489	HMDB0002428	165.020	12.94	2.3E-01	6.6E-01	-5.9E-01	1.5E-01	3.1E-02	-8.8E-02	2.0E-01	5.1E-03	-3.0E-01
A_0034	Perillic acid	1256	HMDB0004586	165.092	7.00	-9.1E-03	9.1E-01	4.4E-02	-9.9E-02	-3.5E-01	-2.8E-02	-2.8E-02	1.4E-01	1.7E-01
A_0035	Quinolinic acid	1066	HMDB0000232	166.014	12.43	-2.8E-01	-7.9E-01	1.2E-01	-1.9E-01	3.0E-01	-1.8E-01	1.0E-01	-3.0E-01	-1.1E-01
A_0036	*N*-Acetyltaurine	159864	HMDB0240253	166.018	7.96	-1.0E-01	-1.5E-01	1.4E-01	-2.7E-01	2.9E-01	-4.4E-01	-5.9E-01	-4.9E-01	1.5E-01
A_0038	Uric acid	1175	HMDB0000289	167.021	7.57	-1.5E-02	4.8E-01	-4.6E-01	4.0E-01	4.9E-02	4.4E-01	1.5E-01	-1.2E-01	-4.1E-01
A_0040	Dihydroxyacetone phosphate	668	HMDB0001473	168.991	10.39	-3.2E-01	-8.0E-01	1.1E-01	-1.3E-01	2.5E-01	-1.9E-01	1.3E-01	-3.2E-01	-1.0E-01
A_0041	Glycerol 3-phosphate	439162	HMDB0000126	171.006	9.98	6.4E-01	3.2E-01	4.0E-01	4.0E-01	-1.7E-01	1.2E-02	-1.8E-02	-2.9E-01	2.2E-01
A_0042	Decanoic acid	2969	HMDB0000511	171.139	6.85	4.7E-01	9.9E-03	-6.3E-01	3.0E-01	2.2E-01	-4.4E-02	-1.6E-01	2.1E-01	4.2E-01
A_0043	Isovalerylalanine-1<br>N-Acetylleucine-1<br>N-Acetylisoleucine-1<br>N-Hexanoylglycine-1	129285<br>70912<br>7036275<br>99463	HMDB0000747<br>HMDB0011756<br>HMDB0061684<br>HMDB0000701	172.098	6.96	2.2E-01	-7.8E-01	-2.5E-02	1.2E-01	-4.6E-01	1.1E-01	-2.0E-01	-1.0E-01	2.3E-01
A_0044	Isovalerylalanine-2<br>N-Acetylleucine-2<br>N-Acetylisoleucine-2<br>N-Hexanoylglycine-2	129285<br>70912<br>7036275<br>99463	HMDB0000747<br>HMDB0011756<br>HMDB0061684<br>HMDB0000701	172.098	6.89	1.9E-01	-3.3E-01	-1.4E-02	5.3E-01	3.4E-01	6.2E-01	-3.3E-02	-1.6E-01	2.1E-01
A_0045	Phenol sulphate	74426	HMDB0060015	172.992	9.04	-2.8E-01	-7.9E-01	1.2E-01	-1.9E-01	3.0E-01	-1.8E-01	1.0E-01	-3.0E-01	-1.1E-01
A_0046	*cis*-Aconitic acid	643757	HMDB0000072	173.009	18.08	-1.6E-01	-4.6E-01	-2.5E-01	4.3E-01	-3.1E-01	-1.3E-01	6.0E-01	1.9E-01	-3.6E-02
A_0047	*N*-Acetylasparagine	99715	HMDB0006028	173.056	7.19	-3.4E-01	-7.7E-02	4.5E-01	7.1E-01	6.3E-02	-5.6E-02	7.0E-03	-1.6E-01	-3.7E-01
A_0048	*N*-Acetylaspartic acid	65065	HMDB0000812	174.041	11.68	3.0E-01	1.9E-01	-4.8E-01	3.9E-01	4.6E-01	-2.4E-01	4.4E-01	-1.3E-02	-1.7E-01
A_0049	Ascorbic acid	54670067	HMDB0000044	175.025	7.26	-2.4E-01	1.1E-01	3.0E-01	6.3E-01	-1.5E-01	-3.4E-01	-4.2E-02	5.1E-01	-2.0E-01
A_0050	Allantoic acid	203	HMDB0001209	175.046	7.41	-3.0E-01	-2.0E-01	2.0E-01	-3.7E-01	-5.4E-01	4.7E-01	-3.4E-01	-2.6E-01	2.1E-02
A_0051	Homovanillic acid<br>Hydroxyphenyllactic acid	1738<br>9378	HMDB0000118<br>HMDB0000755	181.051	7.09	-4.3E-01	-5.8E-01	-2.8E-01	4.6E-01	-2.8E-02	3.1E-01	2.8E-01	-6.2E-02	-9.3E-02
A_0052	3-Phosphoglyceric acid	439183	HMDB0000807	184.985	14.58	-6.0E-02	3.7E-01	-5.9E-01	3.7E-01	-2.9E-01	-3.8E-01	-2.7E-01	-2.0E-01	1.9E-01
A_0054	*N*-Acetylglutamine	25561	HMDB0006029	187.072	6.95	4.7E-01	1.6E-02	-1.5E-01	7.2E-01	-1.0E-01	1.6E-02	-2.8E-01	-3.2E-01	-1.9E-01
A_0055	Azelaic acid	2266	HMDB0000784	187.098	10.04	-6.9E-01	-5.8E-01	-4.6E-02	-1.2E-01	-1.1E-01	-1.9E-01	3.3E-01	5.3E-02	5.3E-02
A_0056	*N*-Acetylglutamic acid	70914	HMDB0001138	188.057	10.76	-9.4E-02	6.4E-01	6.4E-01	1.1E-01	-4.8E-02	2.4E-01	1.5E-02	2.6E-01	2.1E-01
A_0057	*N*-Acetylmethionine	448580	HMDB0011745	190.055	6.97	-5.6E-01	-4.9E-01	-2.6E-01	-4.1E-01	-2.0E-01	-1.9E-01	2.7E-01	1.5E-01	2.0E-01
A_0059	Citric acid	311	HMDB0000094	191.020	17.52	-1.2E-01	-3.8E-01	-9.3E-02	6.9E-01	-2.7E-01	-2.3E-01	3.6E-01	2.5E-01	1.8E-01
A_0060	Quinic acid	6508	HMDB0003072	191.056	7.07	-3.6E-01	-6.9E-01	2.4E-01	-9.1E-02	2.0E-01	-1.1E-01	4.0E-02	4.2E-01	3.2E-01
A_0061	Phenaceturic acid	68144	HMDB0000821	192.066	7.05	-3.8E-01	-8.1E-01	7.2E-02	2.1E-01	-2.5E-01	-2.0E-01	7.3E-02	-3.8E-02	2.0E-01
A_0062	Galacturonic acid-1<br>Glucuronic acid-1	439215<br>94715	HMDB0002545<br>HMDB0000127	193.035	7.09	-3.2E-01	-3.9E-02	4.5E-01	1.2E-01	1.3E-01	-7.9E-01	1.5E-01	9.3E-02	-5.7E-02
A_0063	Galacturonic acid-2<br>Glucuronic acid-2	439215<br>94715	HMDB0002545<br>HMDB0000127	193.035	6.96	5.2E-01	-4.8E-01	9.0E-02	6.1E-02	-3.1E-01	-5.7E-01	-2.4E-01	7.0E-03	-1.2E-01
A_0064	Gluconic acid	10690	HMDB0000625	195.051	7.04	-2.8E-01	3.3E-01	6.7E-01	-3.0E-01	2.0E-01	-1.4E-01	4.1E-01	2.3E-01	3.6E-02
A_0066	Lauric acid	3893	HMDB0000638	199.170	6.61	1.4E-01	5.7E-01	-7.5E-01	-1.0E-01	-1.1E-02	-1.7E-01	1.6E-01	1.4E-01	-1.8E-02
A_0067	3-Indoxylsulfuric acid	10258	HMDB0000682	212.002	8.07	-2.6E-01	-8.3E-01	1.2E-01	1.3E-01	-2.9E-01	-2.7E-01	1.9E-01	-3.4E-02	1.1E-01
A_0068	Pantothenic acid	6613	HMDB0000210	218.104	6.61	-4.8E-01	1.4E-01	6.3E-01	-5.5E-02	2.4E-01	2.1E-01	1.3E-01	-3.1E-01	3.7E-01
A_0069	Ethyl glucuronide	18392195	HMDB0010325	221.067	6.70	2.1E-01	-4.3E-01	5.2E-01	-2.4E-01	3.6E-02	-5.6E-01	-2.8E-01	-1.2E-01	-1.9E-01
A_0070	Myristoleic acid	5281119	HMDB0002000	225.186	6.47	2.7E-01	2.2E-01	-8.3E-01	2.9E-01	5.7E-03	-5.9E-02	3.0E-01	-1.3E-02	5.0E-02
A_0071	Myristic acid	11005	HMDB0000806	227.202	6.44	3.4E-01	2.0E-01	-7.3E-01	2.4E-01	2.6E-01	-2.8E-01	2.9E-01	9.4E-02	-1.1E-01
A_0072	Ribose 5-phosphate	439167	HMDB0001548	229.012	8.84	3.9E-02	-6.5E-01	1.3E-01	2.1E-01	-7.0E-03	-3.1E-01	-4.1E-01	5.1E-01	-4.4E-03
A_0073	Ribulose 5-phosphate	439184	HMDB0000618	229.012	9.16	-8.6E-01	8.1E-02	-4.2E-01	3.0E-02	-2.3E-01	1.1E-01	-6.2E-02	4.2E-02	-8.4E-02
A_0074	*p*-Coumaric acid sulfate	6070438	HMDB0125166	242.996	12.34	4.9E-01	-6.0E-01	6.5E-02	4.6E-01	-1.9E-01	-3.2E-01	7.7E-02	-2.0E-01	2.6E-02
A_0075	Ascorbate 2-sulfate	54676864		254.981	11.50	-3.2E-03	-2.7E-01	-7.3E-01	3.8E-01	-1.3E-01	-2.5E-01	-1.2E-02	-1.0E-01	4.1E-01
A_0076	Glucose 1-phosphate	65533	HMDB0001586	259.022	8.53	-2.6E-01	-8.5E-01	5.5E-02	9.4E-02	8.6E-02	-3.0E-01	8.9E-02	-3.0E-01	-3.4E-02
A_0077	Glucose 6-phosphate	5958	HMDB0001401	259.023	8.31	2.3E-01	-7.9E-01	1.8E-01	3.4E-01	-1.8E-01	-2.8E-01	-1.9E-03	-2.5E-01	5.0E-03
A_0078	*myo*-Inositol 2-phosphate	160886		259.023	8.80	-6.9E-01	-1.8E-01	-5.3E-01	3.7E-01	9.1E-02	-1.7E-01	-1.9E-02	-1.8E-01	-3.6E-02
A_0079	Fructose 6-phosphate	603	HMDB0000124	259.023	8.39	-8.7E-02	-8.4E-01	7.5E-02	2.4E-01	1.7E-01	-3.0E-01	9.1E-02	-3.1E-01	-2.2E-02
A_0080	*myo*-Inositol 1-phosphate<br>*myo*-Inositol 3-phosphate	107737<br>440194	HMDB0000213<br>HMDB0006814	259.023	8.64	1.0E-01	3.6E-01	-8.1E-01	-8.0E-02	3.1E-01	-2.5E-01	6.9E-02	-1.7E-01	-9.6E-02
A_0081	2,3-Diphosphoglyceric acid	186004	HMDB0001294	264.952	13.99	1.4E-01	5.2E-01	-7.4E-01	1.9E-01	-2.3E-01	-1.8E-01	1.7E-01	1.5E-01	-2.3E-02
A_0082	6-Phosphogluconic acid	91493	HMDB0001316	275.018	11.73	-5.5E-01	-4.6E-01	-1.1E-01	5.3E-01	2.6E-01	-2.0E-01	-1.2E-01	-2.7E-01	1.7E-02
A_0083	Xanthosine	64959	HMDB0000299	283.069	6.56	-7.2E-01	3.6E-01	5.3E-02	2.6E-01	3.5E-01	2.6E-01	2.0E-01	-2.4E-01	-4.5E-02
A_0084	Sedoheptulose 7-phosphate	165007	HMDB0001068	289.033	8.13	-7.7E-01	-6.9E-02	-4.9E-01	1.7E-02	-3.2E-01	-5.4E-02	5.0E-02	2.4E-01	3.2E-02
A_0085	*N*-Acetylglucosamine 6-phosphate	440996	HMDB0001062	300.049	7.79	-7.4E-01	6.0E-01	-1.5E-01	-4.6E-02	-2.3E-01	6.7E-02	6.2E-02	-2.7E-02	-6.7E-02

A_0086	*N* -Acetylglucosamine 1-phosphate	440272	HMDB0001367	300.050	8.09	-8.4E-01	3.8E-01	-2.2E-01	-1.0E-02	-2.1E-01	-1.6E-01	-1.4E-01	7.2E-02	-2.3E-02
A_0087	cCMP<br>2',3'-cCMP	19236<br>68934	HMDB0011691	304.034	6.56	3.8E-01	1.9E-01	-5.7E-01	2.4E-01	4.6E-01	-2.4E-01	4.0E-01	1.2E-02	-1.0E-01
A_0088	*N* -Acetylneuraminic acid	439197	HMDB0000230	308.099	6.23	-5.0E-01	2.2E-01	-3.1E-01	2.7E-01	2.6E-01	-6.5E-03	-5.0E-01	1.6E-01	4.4E-01
A_0089	Ribulose 1,5-diphosphate	123658		308.978	12.33	-7.4E-01	2.3E-01	-4.2E-01	-2.5E-01	2.3E-01	1.9E-01	2.4E-01	1.2E-01	8.2E-03
A_0091	CMP	6131	HMDB0000095	322.045	8.10	6.8E-02	-2.9E-01	-9.2E-03	3.8E-02	-6.2E-01	-5.5E-01	-3.4E-01	3.3E-01	1.2E-02
A_0092	3'-CMP	66535		322.046	8.26	3.0E-01	1.9E-01	-4.8E-01	3.9E-01	4.6E-01	-2.4E-01	4.4E-01	-1.3E-02	-1.7E-01
A_0093	UMP	6030	HMDB0000288	323.029	8.24	4.6E-01	-1.2E-01	4.9E-01	5.8E-02	-1.2E-01	-4.5E-01	-2.8E-01	2.9E-01	-3.9E-01
A_0094	*N* -Glycolylneuraminic acid	440001	HMDB0000833	324.094	6.21	-3.8E-01	1.4E-01	-2.1E-01	4.8E-01	4.3E-01	-1.9E-01	-4.7E-01	-2.4E-01	-2.5E-01
A_0096	Ascorbate 2-glucoside	54693473		337.078	6.17	-3.2E-01	-4.3E-01	-1.5E-01	6.7E-01	2.8E-01	2.1E-01	1.2E-01	7.1E-02	-3.2E-01
A_0097	Fructose 1,6-diphosphate	172313	HMDB0001058	338.989	11.66	-2.8E-01	-7.9E-01	1.2E-01	-1.9E-01	3.0E-01	-1.8E-01	1.0E-01	-3.0E-01	-1.1E-01
A_0099	AMP	6083	HMDB0000045	346.056	7.86	4.9E-01	-1.3E-01	5.5E-01	2.0E-02	8.0E-02	-3.6E-02	-3.6E-01	5.5E-01	9.3E-03
A_0100	IMP	8582	HMDB0000175	347.040	8.04	2.2E-01	-8.0E-01	-9.4E-02	4.4E-01	5.2E-02	-7.0E-02	-6.2E-02	-2.8E-01	-1.1E-01
A_0101	GMP	6804	HMDB0001397	362.051	7.75	4.9E-01	-3.0E-02	5.8E-01	2.6E-01	6.8E-03	-3.4E-01	-3.3E-01	3.4E-01	-9.1E-02
A_0102	NADPH_divalent	5884	HMDB0000221	371.536	9.36	2.0E-01	-1.3E-02	7.8E-01	1.9E-01	-5.7E-02	5.0E-01	-9.8E-02	-2.1E-01	-7.4E-02
A_0103	CoA_divalent	87642	HMDB0001423	382.550	8.86	3.0E-01	1.4E-01	3.5E-01	4.8E-01	-5.2E-01	3.5E-01	-3.6E-01	9.3E-02	-7.2E-02
A_0105	FAD_divalent	643975	HMDB0001248	391.571	6.76	-5.7E-01	-4.0E-01	-1.8E-01	3.8E-01	-3.9E-01	-1.9E-01	-1.7E-01	3.3E-01	-1.5E-01
A_0108	UDP	6031	HMDB0000295	402.995	9.59	1.6E-01	4.9E-01	3.6E-01	4.4E-01	2.8E-02	-6.0E-01	1.4E-01	1.5E-01	3.2E-02
A_0109	Acetyl CoA_divalent	444493	HMDB0001206	403.557	8.62	5.5E-01	-3.2E-01	1.9E-01	4.4E-01	-5.4E-01	-1.6E-01	-1.4E-01	-6.0E-02	1.5E-01
A_0110	Cholic acid	221493	HMDB0000619	407.281	5.96	-5.9E-01	1.5E-01	-4.2E-01	-2.5E-01	-5.9E-01	-7.4E-02	1.3E-01	2.6E-02	1.2E-01
A_0111	Propionyl CoA_divalent	92753	HMDB0001275	410.563	8.51	2.9E-01	-4.0E-01	-4.1E-01	-1.1E-02	3.1E-01	-2.8E-01	-5.4E-01	2.5E-01	2.6E-01
A_0112	Isobutyryl CoA_divalent	3036931	HMDB0001243	417.572	8.42	5.5E-01	-3.2E-01	1.9E-01	4.4E-01	-5.4E-01	-1.6E-01	-1.4E-01	-6.0E-02	1.5E-01
A_0113	Thiamine diphosphate	1132	HMDB0001372	423.030	6.82	5.9E-01	1.2E-01	3.3E-01	2.2E-01	-3.4E-01	4.1E-02	3.4E-01	9.3E-02	5.0E-01
A_0115	ADP	6022	HMDB0001341	426.023	9.04	6.1E-01	1.1E-01	3.0E-01	5.2E-01	-1.2E-01	-3.9E-01	-7.9E-02	2.2E-01	2.1E-01
A_0116	GDP	8977	HMDB0001201	442.018	8.86	2.1E-01	4.4E-01	3.9E-01	3.8E-01	9.7E-02	-4.9E-01	4.3E-01	1.1E-01	-1.5E-01
A_0117	Adenylosuccinic acid	447145	HMDB0000536	462.067	11.09	-7.5E-01	-4.7E-01	2.4E-01	4.2E-02	2.4E-01	-1.5E-01	-1.4E-01	2.4E-01	1.5E-02
A_0121	UTP	6133	HMDB0000285	482.961	10.24	3.0E-01	4.9E-01	6.4E-02	5.7E-01	-1.6E-03	-4.7E-01	1.4E-01	-3.3E-01	4.7E-02
A_0122	CDP-choline	13804	HMDB0001413	487.101	5.89	-7.4E-01	3.0E-01	2.3E-01	2.0E-01	2.4E-01	-2.2E-01	3.9E-01	2.7E-02	-1.2E-01
A_0124	ATP	5957	HMDB0000538	505.990	9.67	4.8E-01	2.4E-01	3.3E-02	6.1E-01	-1.5E-01	-4.6E-01	7.3E-02	-8.6E-02	2.9E-01
A_0125	Taurocholic acid	6675	HMDB0000036	514.285	5.85	3.6E-01	-1.2E-02	-4.9E-01	-6.8E-01	-1.3E-01	-5.2E-02	-1.9E-01	1.3E-01	3.2E-01
A_0126	GTP	6830	HMDB0001273	521.984	9.45	2.9E-01	2.2E-01	1.4E-01	1.4E-01	-3.8E-02	-7.6E-01	4.9E-01	3.3E-02	9.5E-02
A_0127	ADP-ribose	445794	HMDB0001178	558.065	7.26	5.3E-01	-6.6E-01	2.0E-01	-4.2E-02	-2.1E-01	-2.4E-01	-1.5E-01	-3.3E-01	1.1E-01
A_0128	UDP-galactose<br>UDP-glucose	23724458<br>8629	HMDB0000302<br>HMDB0000286	565.048	7.36	-4.9E-01	9.0E-02	1.7E-01	5.7E-01	3.9E-02	3.5E-02	-4.8E-01	3.8E-01	1.4E-01
A_0129	UDP-glucuronic acid	17473	HMDB0000935	579.028	9.17	6.0E-01	-2.8E-01	1.8E-01	4.4E-01	-5.0E-01	-2.0E-01	-1.5E-01	-6.3E-02	1.3E-01
A_0130	GDP-fucose<br>ADP-glucose	10918995<br>16500	HMDB0001095<br>HMDB0006557	588.076	7.11	2.8E-01	3.3E-01	1.4E-01	7.8E-01	-3.0E-01	-6.8E-02	1.4E-01	7.9E-02	2.6E-01
A_0131	GDP-mannose<br>GDP-glucose	18396<br>46173703	HMDB0001163<br>HMDB0003351	604.070	7.09	-1.3E-01	-7.2E-03	-5.3E-02	7.1E-01	9.6E-02	6.1E-02	-4.2E-01	3.0E-01	4.4E-01
A_0132	UDP-*N* -acetylgalactosamine<br>UDP-*N* -acetylglucosamine	23724461<br>445675	HMDB0000304<br>HMDB0000290	606.075	7.21	-5.6E-01	2.6E-01	5.0E-01	1.7E-01	1.2E-01	-9.4E-02	-2.4E-01	5.0E-01	9.5E-02
A_0133	CMP-*N* -acetylneuraminate	448209	HMDB0001176	613.145	7.11	-5.6E-02	-2.8E-01	6.2E-01	4.7E-01	-4.1E-01	4.0E-03	3.0E-01	9.6E-02	2.2E-01
A_0134	NAD^+	5893	HMDB0000902	662.102	5.70	6.6E-01	4.8E-01	4.2E-01	2.5E-01	5.3E-02	2.5E-02	1.1E-01	-2.3E-01	1.3E-01
A_0135	NADH	439153	HMDB0001487	664.118	6.99	5.4E-01	1.1E-01	7.1E-01	1.7E-01	-3.5E-01	4.6E-02	-6.5E-02	-9.6E-02	-1.7E-01
A_0136	3'-Dephospho CoA	444485	HMDB0001373	686.143	6.85	-7.6E-01	3.1E-01	4.6E-01	6.4E-02	2.0E-01	8.4E-02	-2.4E-02	-2.5E-01	-1.1E-01
A_0137	$NADP^+$	5886	HMDB0000217	742.069	7.93	7.2E-01	3.0E-01	4.7E-01	3.9E-01	-1.1E-01	-1.3E-02	-8.9E-02	3.0E-02	-1.9E-02
C_0001	Trimethylamine	1146	HMDB0000906	60.081	4.73	-4.2E-01	-8.0E-01	1.5E-01	-6.8E-02	3.2E-01	-7.2E-02	1.5E-01	1.6E-01	7.8E-02
C_0002	Urea	1176	HMDB0000294	61.040	14.62	-6.6E-01	-2.2E-01	-1.5E-01	2.4E-01	-2.3E-01	-4.8E-01	-1.7E-01	-3.5E-01	4.6E-02
C_0003	Ethanolamine	700	HMDB0000149	62.060	5.09	-6.6E-01	5.3E-01	3.2E-01	-1.7E-01	-2.0E-01	5.7E-02	1.7E-01	-2.6E-01	7.0E-02
C_0004	Aminoacetone	215	HMDB0002134	74.060	5.47	-7.4E-01	2.9E-01	1.9E-01	-3.6E-01	4.3E-02	8.3E-02	-3.9E-01	1.6E-01	1.4E-01
C_0005	Gly	750	HMDB0000123	76.039	6.53	-5.0E-01	4.9E-01	-1.6E-01	2.5E-01	9.2E-02	-4.9E-01	-3.7E-01	-9.2E-02	-1.6E-01
C_0006	Trimethylamine *N* -oxide	1145	HMDB0000925	76.076	5.27	-8.7E-02	-4.3E-01	-3.2E-01	6.5E-01	5.0E-01	-9.5E-02	1.4E-02	-2.8E-02	1.8E-01
C_0007	Piperidine	8082		86.097	5.54	-5.2E-01	2.1E-01	-2.9E-01	-1.0E-01	-6.5E-01	-2.1E-01	3.3E-01	-1.6E-01	-3.7E-02
C_0008	Putrescine	1045	HMDB0001414	89.107	3.84	-3.9E-01	-6.9E-01	2.5E-01	7.0E-02	8.4E-02	4.1E-01	9.9E-02	3.5E-01	-3.0E-02
C_0009	Sarcosine	1088	HMDB0000271	90.055	7.35	-4.3E-01	2.4E-01	-6.7E-01	3.8E-01	-1.2E-01	-3.2E-02	2.1E-01	-2.8E-01	-1.6E-01
C_0010	Ala	602	HMDB0000161,H	90.055	7.02	-4.6E-01	1.6E-01	3.3E-01	6.1E-01	-7.1E-02	4.8E-01	1.1E-01	1.9E-01	-3.7E-02
C_0011	β-Ala	239	HMDB0000056	90.055	5.81	-5.9E-01	6.2E-01	3.5E-01	-9.7E-02	1.3E-01	3.2E-01	3.3E-02	2.8E-02	-1.2E-01
C_0012	Dimethylaminoethanol	7902	HMDB0032231	90.091	5.51	-9.1E-03	2.5E-01	5.9E-01	-8.6E-02	1.2E-01	6.0E-01	3.9E-01	1.7E-01	1.3E-01
C_0013	Glycerol	753	HMDB0000131	93.055	15.10	-2.6E-01	2.2E-01	1.2E-01	3.7E-01	6.4E-01	4.8E-02	-7.1E-02	1.8E-01	5.3E-01
C_0014	Aniline	6115	HMDB0003012	94.065	5.87	-2.1E-01	-3.4E-01	2.5E-01	-3.6E-01	-2.8E-01	-4.2E-01	3.5E-01	5.0E-01	-1.5E-01
C_0015	Cyclohexylamine	7965		100.112	5.87	-8.3E-01	-2.5E-01	1.1E-01	-1.0E-03	-2.0E-01	-4.2E-01	-2.6E-02	-5.1E-02	-1.2E-01
C_0016	Homoserinelactone	73509		102.056	5.65	1.5E-01	-8.6E-01	2.3E-01	1.5E-01	-1.2E-01	-2.6E-01	-9.7E-03	-2.9E-01	1.0E-02
C_0017	3-Aminoisobutyric acid	64956	HMDB0003911	104.070	6.17	-5.0E-01	4.4E-01	6.3E-01	-1.5E-01	2.5E-01	4.1E-03	-1.4E-01	-1.5E-02	2.3E-01
C_0018	2-Aminoisobutyric acid<br>2-Aminobutyric acid	6119<br>6657	HMDB0001906<br>HMDB0000452	104.070	7.44	-1.8E-01	8.2E-01	4.9E-01	2.0E-01	-7.1E-02	2.9E-02	-3.4E-03	-1.5E-02	-9.4E-02
C_0019	GABA	119	HMDB0000112	104.070	6.07	-2.7E-01	-7.9E-01	2.0E-01	8.3E-02	3.2E-01	-3.3E-01	-8.5E-02	-1.9E-01	1.0E-01
C_0020	*N* ,*N* -Dimethylglycine	673	HMDB0000092	104.071	8.34	-2.7E-01	-3.7E-02	-2.6E-01	7.5E-01	-1.4E-01	3.9E-01	-2.5E-01	-2.2E-01	-9.3E-02
C_0021	3-Aminobutyric acid	10932		104.071	6.25	-5.1E-02	5.6E-01	-3.3E-01	-1.3E-01	-6.0E-01	-3.2E-01	-1.1E-01	-2.6E-01	-1.2E-01
C_0022	Choline	305	HMDB0000097	104.107	5.46	-5.9E-01	4.6E-01	3.2E-01	-1.1E-01	-2.6E-01	3.8E-01	2.5E-01	-2.2E-01	-7.6E-02
C_0023	Ser	617	HMDB0000187,H	106.050	7.71	-2.4E-01	2.8E-01	6.9E-01	2.1E-01	1.9E-01	-3.8E-01	-3.9E-01	-5.9E-02	-1.2E-01
C_0024	Diethanolamine	8113	HMDB0004437	106.086	6.03	1.3E-01	6.6E-01	1.8E-01	-1.7E-01	4.7E-01	-2.8E-01	-4.0E-01	-1.5E-01	5.5E-02
C_0025	Hypotaurine	107812	HMDB0000965	110.027	12.77	-1.5E-01	2.5E-01	6.4E-01	-3.1E-01	3.0E-01	2.3E-01	4.6E-01	-7.6E-02	2.2E-01
C_0027	Histamine	774	HMDB0000870	112.087	3.89	3.0E-01	1.9E-01	-4.8E-01	3.9E-01	4.6E-01	-2.4E-01	4.4E-01	-1.3E-02	-1.7E-01
C_0028	Uracil	1174	HMDB0000300	113.034	15.13	-9.2E-01	3.7E-02	-2.3E-01	-9.0E-02	-1.1E-01	-1.3E-01	2.5E-01	-3.8E-03	-5.7E-02
C_0029	Creatinine	588	HMDB0000562	114.066	5.78	-5.4E-01	-7.7E-01	8.7E-02	4.0E-02	-1.2E-01	-2.4E-01	1.3E-01	-1.1E-01	5.4E-02
C_0030	Pro	614	HMDB0000162,H	116.071	8.24	-8.7E-01	2.7E-01	-1.4E-01	2.9E-01	1.5E-01	1.3E-01	9.0E-02	5.2E-02	8.9E-02
C_0031	Guanidoacetic acid	763	HMDB0000128	118.061	6.46	-1.5E-01	3.4E-01	4.2E-01	1.6E-01	-2.5E-02	-7.1E-01	2.4E-01	-2.2E-01	-2.0E-01
C_0032	Val	1182	HMDB0000883	118.086	7.69	-9.1E-01	2.9E-01	-8.4E-02	7.3E-02	1.8E-01	1.5E-01	-1.0E-01	-8.2E-02	5.4E-02
C_0033	Betaine	247	HMDB0000043	118.086	8.56	-1.5E-01	2.0E-01	7.3E-01	2.4E-01	-3.0E-01	3.4E-01	-2.2E-01	-2.0E-01	-2.4E-01
C_0034	Homoserine	12647	HMDB0000719	120.065	7.75	-1.7E-01	1.8E-01	5.4E-01	1.5E-01	-4.0E-01	-4.6E-01	-1.8E-01	-4.6E-01	8.9E-02
C_0035	Thr	6288	HMDB0000167	120.065	8.06	-8.2E-01	2.5E-01	1.4E-01	1.9E-01	3.3E-01	-1.9E-01	-2.6E-01	-8.5E-03	-5.1E-02
C_0036	Betaine aldehyde_+H_2O	249	HMDB0001252	120.102	5.90	-8.4E-01	-2.3E-01	2.8E-01	-2.2E-01	1.1E-01	-1.6E-01	2.0E-02	-2.7E-01	-2.6E-02
C_0037	Anserine_divalent	112072	HMDB0000194	121.068	5.44	-5.2E-01	-3.7E-02	-1.8E-01	3.9E-01	5.0E-01	3.0E-01	-4.6E-01	1.8E-02	3.9E-02
C_0038	Cys	594	HMDB0000574,H	122.027	8.59	-1.6E-01	1.7E-01	-5.3E-01	2.3E-01	5.3E-01	1.2E-01	6.0E-02	2.8E-01	4.9E-01
C_0039	Nicotinamide	936	HMDB0001406	123.055	5.87	-8.8E-01	-3.2E-01	-1.0E-02	-1.1E-02	-2.5E-01	-1.5E-01	-9.2E-02	1.5E-01	-1.0E-01
C_0040	Picolinic acid	1018	HMDB0002243	124.038	13.21	-7.2E-01	-3.3E-01	1.4E-02	-1.0E-01	-2.7E-01	7.4E-02	3.2E-01	3.7E-01	-2.0E-01
C_0041	Taurine	1123	HMDB0000251	126.022	15.10	-3.5E-01	4.8E-01	-3.4E-01	3.0E-01	-1.6E-01	-1.4E-01	-5.8E-01	2.1E-01	-1.4E-01
C_0042	1-Methylhistamine	3614	HMDB0000898	126.102	4.01	3.4E-02	-8.9E-01	-1.3E-02	-3.2E-01	1.1E-01	-1.6E-01	-2.9E-02	8.1E-02	2.4E-01
C_0043	3-Hydroxy-2-methyl-4-pyrone	8369	HMDB0030776	127.038	15.15	-4.4E-01	-5.4E-01	2.0E-01	3.6E-01	3.7E-01	-9.5E-02	2.0E-02	-4.2E-01	1.2E-01
C_0044	Imidazole-4-acetic acid	96215	HMDB0002024	127.050	6.31	8.1E-02	1.7E-01	-1.2E-01	3.6E-01	-5.8E-01	-1.3E-01	1.1E-01	3.6E-01	5.6E-01
C_0046	5-Amino-3,4-dihydro-2H-pyrrole-2-carboxylic acid	13894665		129.066	6.83	-6.1E-02	-3.9E-01	1.5E-01	-6.9E-02	-7.8E-01	1.1E-01	4.0E-01	1.0E-02	1.8E-01
C_0047	4-Oxopyrrolidine-2-carboxylic acid	107541		130.050	8.35	-8.4E-02	6.9E-01	2.9E-01	4.3E-01	5.6E-02	-1.4E-01	2.4E-01	-4.2E-01	-5.4E-02
C_0048	Pipecolic acid	439227	HMDB0000070,H	130.086	7.88	-3.0E-01	4.2E-02	-3.7E-01	2.2E-01	-6.5E-01	-4.9E-01	-2.2E-02	-8.4E-02	2.4E-01

C_0049	Hydroxyproline	5810	HMDB0000725	132.065	9.06	-2.1E-01	4.4E-01	7.5E-01	1.1E-01	1.6E-01	-7.3E-02	1.2E-01	3.7E-01	6.9E-02
C_0050	3-Hydroxyproline	150779	HMDB0002113	132.066	8.27	3.9E-01	-4.2E-03	-4.1E-01	-7.2E-01	4.5E-03	-6.9E-03	-2.1E-01	1.2E-01	3.1E-01
C_0051	Creatine	586	HMDB0000064	132.076	6.89	2.3E-01	3.1E-01	-5.7E-01	3.7E-01	3.5E-01	1.3E-01	4.9E-01	1.6E-03	4.2E-02
C_0052	Ile	791	HMDB0000172	132.102	7.83	-9.2E-01	2.2E-01	-1.1E-01	6.6E-02	1.3E-01	2.2E-01	-7.3E-02	-7.6E-02	1.1E-01
C_0053	Leu	857	HMDB0000687	132.102	7.90	-9.6E-01	1.4E-01	-1.1E-01	1.1E-02	1.7E-01	4.7E-02	-1.0E-01	-7.9E-02	6.9E-02
C_0054	Gly-Gly	11163	HMDB0011733	133.060	6.56	-7.0E-01	5.6E-01	-6.9E-02	-2.5E-01	7.7E-02	8.2E-02	-1.7E-01	2.8E-01	-9.3E-02
C_0055	Asn	236	HMDB0000168,H	133.061	8.05	-7.9E-01	-2.0E-01	-1.7E-01	1.3E-01	3.4E-01	-3.7E-01	-8.5E-02	-1.4E-01	-1.1E-01
C_0056	Ornithine	389	HMDB0000214,H	133.097	5.43	-4.4E-01	2.9E-01	5.4E-01	-3.1E-01	1.7E-01	-3.5E-04	4.3E-01	-2.7E-01	2.1E-01
C_0057	Thiaproline	9934		134.027	10.33	-5.2E-02	3.9E-01	-5.8E-01	5.0E-01	2.0E-01	2.3E-01	2.5E-01	1.1E-01	3.1E-01
C_0058	Asp	424	HMDB0000191,H	134.045	8.78	1.0E-01	7.3E-01	6.1E-01	1.5E-01	1.5E-01	-1.5E-01	2.1E-02	7.5E-02	9.2E-02
C_0059	Adenine	190	HMDB0000034	136.062	6.02	-8.9E-01	2.2E-01	-1.7E-02	7.6E-02	-2.3E-01	2.7E-01	1.1E-01	-1.3E-01	6.7E-02
C_0060	Hypoxanthine	790	HMDB0000157	137.046	8.56	-8.8E-01	5.8E-02	-1.9E-01	-1.2E-01	-3.6E-01	-6.0E-02	1.8E-01	-1.5E-03	5.1E-02
C_0061	1-Methylnicotinamide	457	HMDB0000699	137.070	5.83	-5.6E-01	1.2E-01	-3.2E-01	1.8E-01	-1.9E-01	3.9E-01	-3.0E-01	-5.0E-01	-2.2E-02
C_0063	Trigonelline	5570	HMDB0000875	138.055	8.01	-1.9E-01	2.9E-01	4.8E-01	6.2E-01	2.6E-01	-3.5E-01	-1.9E-01	-1.1E-01	1.8E-01
C_0065	γ-Glu-Lys_divalent	65254	HMDB0029154	138.582	6.70	3.7E-01	1.1E-01	4.4E-02	-3.0E-01	5.4E-01	7.1E-02	2.9E-01	-9.3E-02	-6.0E-01
C_0066	Nicotinamide *N*-oxide	72661	HMDB0002730	139.049	15.07	-4.4E-01	-6.4E-01	1.4E-01	4.0E-02	2.6E-01	3.3E-02	1.5E-01	4.9E-01	2.1E-01
C_0067	Urocanic acid	736715	HMDB0000301	139.050	6.49	5.2E-01	1.3E-01	-6.6E-01	-3.0E-01	3.3E-01	-1.7E-01	1.4E-01	8.5E-02	1.2E-01
C_0068	1-Methyl-4-imidazoleacetic acid	75810	HMDB0002820	141.065	6.46	-8.6E-01	-3.0E-01	-2.1E-01	1.7E-01	-5.3E-02	4.8E-02	-9.1E-02	-3.0E-02	2.7E-01
C_0069	4-Guanidinobutyric acid	500	HMDB0003464	146.092	6.49	3.0E-01	1.9E-01	-4.8E-01	3.9E-01	4.6E-01	-2.4E-01	4.4E-01	-1.3E-02	-1.7E-01
C_0070	γ-Butyrobetaine	134	HMDB0001161	146.117	6.36	-5.8E-01	4.9E-01	-9.3E-02	1.6E-01	-3.2E-01	-6.1E-03	2.8E-01	2.5E-01	3.7E-01
C_0071	Spermidine	1102	HMDB0001257	146.165	3.70	-4.5E-01	-3.2E-01	1.2E-02	2.2E-02	-6.6E-01	2.1E-01	-8.8E-02	-1.7E-01	-4.3E-01
C_0072	Gln	738	HMDB0000641,H	147.076	8.22	-2.5E-01	9.6E-02	-4.5E-01	6.0E-01	-1.7E-01	-4.0E-01	-8.9E-02	-4.0E-01	-7.0E-02
C_0073	Lys	866	HMDB0000182,H	147.113	5.47	-7.4E-01	4.9E-01	8.6E-02	-3.3E-02	1.5E-01	-9.4E-02	-3.4E-01	2.4E-01	2.9E-02
C_0074	2-Methylthiazolidine-4-carboxylic acid	160736	HMDB0246657	148.042	10.48	1.1E-01	-1.3E-01	4.3E-01	-3.9E-01	3.0E-01	-4.2E-01	-4.2E-01	-3.7E-01	2.3E-01
C_0075	Glu	611	HMDB0000148,H	148.060	8.35	-3.4E-02	6.5E-01	5.3E-01	3.3E-01	1.3E-01	-2.8E-01	7.2E-02	-3.0E-01	7.4E-02
C_0076	Isoglutamic acid	73064		148.061	7.11	2.7E-01	2.0E-02	-4.0E-01	3.4E-02	-6.1E-01	3.8E-01	4.4E-01	9.1E-02	-2.3E-01
C_0077	Met	876	HMDB0000696	150.058	8.19	-8.4E-01	3.0E-01	-8.0E-02	-1.5E-01	1.8E-02	-3.3E-01	-1.5E-01	2.1E-01	1.1E-02
C_0078	Triethanolamine	7618		150.112	6.46	3.8E-01	2.5E-01	3.9E-02	-7.2E-01	1.2E-01	-2.4E-02	-5.1E-03	-6.1E-02	5.0E-01
C_0079	Guanine	764	HMDB0000132	152.056	6.52	3.9E-01	-4.2E-03	-4.1E-01	-7.2E-01	4.5E-03	-6.9E-03	-2.1E-01	1.2E-01	3.1E-01
C_0080	Xanthine	1188	HMDB0000292	153.041	13.72	-9.0E-01	1.5E-01	-9.0E-02	-4.4E-02	-2.7E-01	1.7E-01	1.8E-01	-7.7E-02	-1.2E-01
C_0081	N^1-Methyl-4-pyridone-5-carboxamide	440810	HMDB0004194	153.066	13.09	-6.7E-01	2.3E-01	-3.3E-01	2.5E-01	-3.1E-01	4.1E-01	-1.8E-01	-1.2E-01	1.2E-01
C_0082	His	773	HMDB0000177	156.077	5.79	-6.8E-01	3.3E-01	1.1E-01	4.6E-01	-1.4E-01	1.9E-01	-3.3E-01	1.2E-01	-1.6E-01
C_0083	Imidazolelactic acid	793		157.060	6.88	-6.9E-01	-1.8E-01	8.6E-02	-2.4E-01	-2.6E-01	-2.2E-01	-3.6E-03	2.9E-01	-4.8E-01
C_0084	N^6-Methyllysine	164795	HMDB0002038	161.128	5.63	-9.4E-02	-1.6E-02	-1.3E-01	1.9E-01	-8.9E-01	-2.8E-01	1.9E-01	-1.7E-01	6.2E-02
C_0085	Glutamic acid γ-methyl ester<br>O- Acetylhomoserine<br>2-Aminoadipic acid	68662<br>439389<br>92136	HMDB0061715<br><br>HMDB0000510	162.076	8.36	-6.6E-01	3.5E-02	4.9E-01	-2.4E-01	4.5E-01	-1.7E-01	1.6E-01	8.2E-02	-3.7E-02
C_0086	Carnitine	85	HMDB0000062	162.112	6.65	-6.6E-01	2.0E-01	6.2E-02	2.8E-02	-5.7E-01	2.8E-01	3.0E-01	-6.7E-02	1.2E-01
C_0087	Methionine sulfoxide	158980	HMDB0002005	166.053	8.91	-7.4E-01	-1.5E-01	7.5E-02	-3.5E-01	-4.2E-01	-2.4E-01	-2.0E-01	-1.8E-01	1.4E-02
C_0088	Phe	994	HMDB0000159	166.086	8.43	-9.4E-01	8.8E-03	-2.4E-01	-3.5E-02	1.8E-01	5.9E-02	-2.2E-02	-1.0E-01	8.6E-02
C_0089	Taurocyamine	68340	HMDB0003584	168.044	15.12	-4.9E-01	1.3E-01	6.1E-01	6.6E-02	-2.2E-01	-3.0E-01	4.8E-01	1.8E-02	-3.0E-02
C_0090	Tyr-Arg_divalent	123804		169.595	6.07	-3.7E-01	6.5E-02	-5.8E-01	-1.1E-02	1.2E-01	7.1E-01	6.0E-02	-1.9E-02	9.1E-02
C_0091	1-Methylhistidine<br>3-Methylhistidine	92105<br>64969	HMDB0000001<br>HMDB0000479	170.092	5.92	3.5E-02	-7.7E-03	-1.7E-01	5.5E-01	-5.4E-01	4.5E-01	3.5E-01	-1.9E-01	-1.2E-01
C_0092	Arg	6322	HMDB0000517,H	175.119	5.65	3.1E-01	-3.0E-01	-5.5E-01	-2.2E-01	5.4E-01	-2.9E-01	2.8E-01	-1.1E-01	-1.9E-02
C_0093	Guanidinosuccinic acid	439918	HMDB0003157	176.067	7.77	-6.5E-01	-2.0E-01	-2.8E-01	3.7E-01	3.4E-01	-3.7E-01	-2.1E-01	-8.3E-02	-1.5E-01
C_0094	Citrulline	9750	HMDB0000904	176.103	8.42	7.3E-02	5.4E-01	-3.2E-01	6.2E-02	-3.8E-01	-3.2E-01	4.4E-01	2.1E-01	3.4E-01
C_0095	Argininic acid	160437	HMDB0003148	176.103	7.09	-1.8E-01	-3.4E-01	5.6E-01	-2.6E-01	3.8E-01	-1.7E-01	2.9E-01	-4.3E-01	1.9E-01
C_0096	Serotonin	5202	HMDB0000259	177.103	6.83	7.1E-02	5.1E-01	-5.7E-01	-2.4E-01	8.2E-02	-1.4E-01	-3.3E-01	1.9E-02	-4.7E-01
C_0097	Gluconolactone	7027	HMDB0000150	179.055	15.56	-4.0E-01	3.6E-01	6.8E-01	-2.0E-01	2.4E-01	-1.8E-01	3.0E-01	1.4E-01	-1.0E-01
C_0098	Glucosamine	439213	HMDB0001514	180.086	7.19	-9.4E-01	-1.3E-01	8.6E-02	9.6E-02	1.8E-01	-3.8E-04	1.4E-01	-5.8E-02	-1.8E-01
C_0099	Theobromine	5429	HMDB0002825	181.070	15.14	1.7E-01	4.7E-01	-2.1E-01	-1.0E-01	2.6E-01	-3.9E-01	-2.4E-02	-6.1E-01	-3.4E-01
C_0100	Tyr	1153	HMDB0000158	182.081	8.51	-9.0E-01	-5.2E-02	-1.1E-01	4.9E-02	2.7E-01	7.1E-03	-1.6E-01	2.6E-01	-5.9E-02
C_0101	Phosphorylcholine	1014	HMDB0001565	184.073	14.22	-5.1E-01	-9.2E-02	2.5E-01	5.1E-01	1.5E-01	2.1E-01	4.1E-01	3.2E-01	-2.8E-01
C_0102	N^8-Acetylspermidine	123689	HMDB0002189	188.176	5.07	1.2E-01	-4.6E-01	-4.2E-02	1.2E-01	6.0E-01	3.8E-01	-2.0E-02	-3.7E-01	3.4E-01
C_0103	*N*-Acetyllysine	92907	HMDB0000446	189.123	7.53	-4.7E-02	6.1E-01	-5.9E-01	2.2E-01	3.3E-01	-1.7E-01	-1.7E-01	-2.4E-01	2.3E-02
C_0104	N^6,N^6,N^6-Trimethyllysine	440120	HMDB0001325	189.159	5.70	-5.1E-01	3.9E-01	-5.9E-01	-6.6E-02	-3.8E-01	-1.9E-01	1.1E-01	8.4E-02	1.7E-01
C_0105	Gly-Asp	97363		191.066	7.61	-9.1E-01	-1.1E-03	1.3E-01	5.5E-03	-6.9E-02	-2.5E-01	-3.8E-02	3.0E-01	3.6E-02
C_0106	H-Asp(Gly-OH)-OH	99717	HMDB0011165	191.067	9.03	1.1E-01	-5.6E-01	6.1E-01	1.5E-01	-8.3E-02	3.9E-01	2.9E-01	1.3E-01	1.2E-01
C_0107	γ-Carboxyglutamic acid	40772	HMDB0041900	192.050	10.61	-6.7E-02	4.8E-01	-4.6E-01	-1.1E-01	-3.0E-02	-2.1E-01	-6.6E-01	-5.3E-02	2.5E-01
C_0108	Caffeine	2519	HMDB0001847	195.087	15.15	-3.8E-01	-5.6E-01	4.6E-01	-1.3E-01	4.0E-01	-7.6E-02	2.9E-01	4.3E-02	2.7E-01
C_0109	*N*-Acetylhistidine	75619		198.087	7.55	-3.1E-01	1.0E-01	-4.2E-02	3.2E-01	-7.1E-02	8.0E-01	-3.3E-01	7.8E-02	-1.6E-01
C_0110	SDMA	169148	HMDB0003334	203.150	6.14	-6.3E-01	2.0E-03	5.2E-01	-2.7E-01	-1.0E-01	-8.5E-02	2.7E-01	2.2E-01	-3.5E-01
C_0111	ADMA	123831	HMDB0001539	203.150	6.04	-6.7E-01	2.3E-01	-2.4E-01	9.4E-03	-5.8E-01	3.3E-02	1.2E-01	-5.3E-03	2.8E-01
C_0112	Spermine	1103	HMDB0001256	203.223	3.66	-3.4E-01	-3.4E-01	2.8E-01	-2.3E-02	-4.5E-01	-9.3E-02	-4.1E-01	1.0E-01	-5.5E-01
C_0113	*O*-Acetylcarnitine	439756	HMDB0000201	204.123	7.02	6.2E-01	-2.3E-01	7.5E-02	6.0E-01	-2.7E-01	-2.9E-01	6.1E-02	6.5E-02	1.8E-01
C_0114	γ-Glu-Gly	165527	HMDB0011667	205.082	9.07	1.9E-01	4.9E-01	4.4E-01	8.8E-03	-1.5E-01	-6.9E-01	1.9E-02	1.4E-01	1.2E-01
C_0115	Trp	1148	HMDB0000929	205.097	8.39	-7.3E-01	3.5E-01	-3.6E-01	2.8E-01	3.1E-01	7.2E-02	-4.8E-02	3.3E-02	1.8E-01
C_0116	Kynurenine	846	HMDB0000684	209.092	7.68	-7.9E-01	-2.6E-01	-3.5E-01	-1.2E-01	-4.3E-02	6.3E-02	2.9E-01	-4.2E-02	2.9E-01
C_0117	Propionylcarnitine	188824	HMDB0000824	218.138	7.22	-7.3E-01	1.8E-01	-4.2E-01	2.7E-01	1.6E-01	1.3E-01	1.9E-01	3.2E-01	-2.5E-02
C_0118	β-Ala-Lys	440638		218.150	5.43	2.3E-01	1.3E-01	5.8E-01	5.0E-01	-1.6E-01	4.6E-01	-2.8E-01	-1.0E-01	1.3E-01
C_0119	*N*-Acetylgalactosamine<br>*N*-Acetylglucosamine<br>*N*-Acetylmannosamine	35717<br>439174<br>439281	HMDB0000853<br>HMDB0000215<br>HMDB0001129	222.096	15.13	-6.3E-01	4.6E-01	1.6E-01	8.6E-02	-2.0E-01	5.1E-02	1.6E-01	-1.2E-01	5.2E-01
C_0120	Cystathionine	834	HMDB0000099	223.075	7.64	2.4E-01	9.8E-02	1.1E-01	5.0E-01	-6.4E-02	7.4E-01	-8.8E-02	-3.2E-01	4.8E-02
C_0122	7,8-Dihydrobiopterin	119055	HMDB0000038	240.109	8.39	3.3E-01	-2.7E-01	3.5E-01	4.4E-01	4.5E-01	1.7E-01	-3.5E-01	2.5E-01	2.8E-01
C_0124	Cytidine	6175	HMDB0000089	244.093	7.46	-7.9E-01	3.0E-01	-2.0E-01	-1.3E-01	-1.4E-01	-4.5E-01	7.3E-02	-7.0E-02	4.4E-02
C_0125	Uridine	6029	HMDB0000296	245.077	15.14	-9.6E-01	-5.3E-02	-5.2E-02	1.4E-02	-1.2E-01	-9.7E-03	2.2E-01	-9.4E-02	-5.5E-02
C_0126	γ-Glu-Val	7015683	HMDB0011172	247.129	9.50	-1.2E-01	4.1E-01	6.8E-01	4.0E-02	-3.5E-01	-4.0E-01	1.0E-01	2.0E-01	1.3E-01
C_0127	Pyridoxamine 5'-phosphate	1053	HMDB0001555	249.063	8.01	-1.0E-01	7.1E-01	4.6E-01	1.9E-01	-2.9E-01	-3.5E-01	1.7E-01	-5.1E-02	-2.9E-02
C_0128	γ-Glu-Thr	53861142	HMDB0029159	249.109	9.51	-1.2E-01	4.4E-01	7.1E-01	4.2E-03	-2.7E-01	-3.3E-01	1.9E-01	1.3E-01	2.3E-01
C_0129	γ-Glu-Cys	123938	HMDB0001049	251.070	9.57	-8.0E-01	1.8E-01	2.4E-01	1.9E-01	3.4E-01	2.2E-01	-3.6E-02	2.4E-01	8.6E-02
C_0130	Glycerophosphocholine	439285	HMDB0000086	258.110	14.89	1.7E-01	1.7E-01	3.8E-01	1.2E-01	6.6E-03	7.0E-01	3.6E-01	-2.5E-01	-3.3E-01
C_0131	γ-Glu-Ile<br>γ-Glu-Leu	22885096<br>151023	HMDB0011170<br>HMDB0011171	261.144	9.64	-3.1E-03	2.2E-01	5.1E-01	-3.4E-01	-1.0E-01	-3.9E-01	4.7E-01	4.1E-01	-1.5E-01
C_0132	γ-Glu-Asp	161197	HMDB0030419	263.087	9.67	6.1E-02	4.2E-01	6.6E-01	-1.6E-01	2.0E-01	-2.9E-02	3.0E-01	-2.7E-01	3.9E-01
C_0133	Thiamine	1130	HMDB0000235	265.112	5.26	-8.9E-01	-4.0E-01	-1.9E-01	1.7E-02	-1.5E-01	2.1E-01	7.3E-02	-2.4E-01	-2.5E-02
C_0134	Adenosine	60961	HMDB0000050	268.104	7.60	-1.6E-01	6.2E-01	-7.3E-02	-2.0E-02	2.2E-01	-3.8E-01	-4.5E-01	4.2E-01	-1.1E-01
C_0135	Inosine	6021	HMDB0000195	269.088	13.72	-8.8E-01	9.9E-02	-2.7E-01	-3.3E-02	-3.5E-01	-3.4E-02	9.3E-02	-3.1E-02	-3.3E-02
C_0136	γ-Glu-Gln	150914	HMDB0011738	276.119	9.68	3.0E-01	1.9E-01	-4.8E-01	3.9E-01	4.6E-01	-2.4E-01	4.4E-01	-1.3E-02	-1.7E-01
C_0137	γ-Glu-Glu	92865	HMDB0011737	277.103	9.75	2.6E-01	-4.0E-01	-2.7E-01	6.6E-02	5.0E-01	-5.3E-01	1.2E-01	3.4E-01	-2.0E-01
C_0138	Saccharopine	160556	HMDB0000279	277.139	8.19	-6.3E-01	-2.9E-02	2.2E-01	-3.3E-01	2.7E-01	-3.1E-01	3.5E-01	-8.7E-02	3.9E-01
C_0139	Guanosine	6802	HMDB0000133	284.099	9.45	-6.0E-01	5.3E-01	-1.1E-01	8.1E-02	-1.0E-01	-4.8E-01	-1.6E-01	2.3E-01	-1.5E-01

C_0140	Ophthalmic acid	7018721	HMDB0005765	290.134	9.83	-1.7E-01	6.1E-01	4.1E-01	-1.1E-01	-2.6E-01	-5.5E-01	-1.2E-02	-1.6E-01	1.5E-01
C_0141	Argininosuccinic acid	16950	HMDB0000052	291.130	7.30	2.7E-01	-1.5E-01	8.3E-02	3.5E-01	1.0E-02	-4.8E-01	5.9E-01	-4.2E-01	1.2E-01
C_0142	5'-Deoxy-5'-methylthioadenosine	439176	HMDB0001173	298.097	7.75	1.2E-01	-7.3E-01	2.1E-01	2.8E-01	1.6E-01	6.0E-04	1.5E-01	2.6E-01	-4.7E-01
C_0143	Glutathione (GSSG)_divalent	65359	HMDB0003337	307.083	9.16	-1.0E-01	-9.2E-02	5.4E-01	5.4E-01	-5.3E-01	4.3E-02	2.0E-01	-1.5E-01	-2.2E-01
C_0144	Glutathione (GSH)	124886	HMDB0000125	308.091	9.84	-6.2E-01	-2.5E-01	3.3E-01	3.6E-01	3.8E-01	2.1E-01	-2.6E-01	2.0E-01	1.2E-01
C_0145	*S*-Methylglutathione	115260		322.107	9.93	-5.7E-01	-2.8E-01	3.8E-01	7.4E-02	3.8E-01	4.7E-01	-2.3E-01	1.6E-01	-5.2E-02
C_0146	NMN	14180	HMDB0000229	335.064	14.30	8.8E-02	8.3E-01	4.1E-01	-1.6E-01	9.2E-02	-1.5E-01	-1.3E-01	-3.2E-02	-2.2E-01
C_0147	Topiramic acid	5284627	HMDB0005034	340.109	15.07	3.7E-01	-2.6E-01	-1.7E-01	-2.1E-01	-2.1E-01	3.5E-02	-1.6E-01	6.0E-01	5.3E-01
C_0148	Thiamine phosphate	1131	HMDB0002666	345.078	8.18	-3.9E-01	-3.5E-01	1.3E-01	2.8E-01	-3.2E-01	1.5E-01	-2.1E-01	6.2E-02	6.8E-01
C_0149	*S*-Lactoylglutathione	440018	HMDB0001066	380.114	10.19	-6.3E-01	-4.3E-01	-3.1E-02	-2.8E-01	1.8E-01	-4.7E-01	1.0E-01	-2.5E-01	-1.3E-01
C_0150	Succinyladenosine	165243	HMDB0000912	384.114	10.67	-7.1E-01	5.5E-02	4.8E-01	2.7E-02	4.4E-01	-1.3E-01	-1.7E-01	-7.9E-02	1.1E-01
C_0151	*S*-Adenosylhomocysteine	439155	HMDB0000939	385.129	6.82	-5.7E-01	3.8E-01	-6.0E-01	2.0E-01	-2.1E-01	-9.6E-02	2.4E-01	-8.3E-02	-1.0E-01
C_0152	*S*-Adenosylmethionine	34755	HMDB0001185	399.144	5.65	-1.9E-01	-8.0E-01	3.5E-01	2.2E-01	1.0E-01	2.6E-01	-2.0E-01	7.5E-02	-1.6E-01
C_0153	Cysteine glutathione disulfide	10455148	HMDB0000656	427.095	8.81	4.1E-01	2.8E-01	-3.3E-01	1.2E-01	5.6E-01	-2.2E-02	2.1E-01	1.3E-01	5.0E-01
C_0154	CDP-ethanolamine	123727	HMDB0001564	447.067	14.82	-3.9E-01	5.5E-02	5.6E-01	2.9E-01	-4.1E-02	-6.1E-01	-8.9E-02	2.3E-01	-4.2E-02
C_0155	5-Methyltetrahydrofolic acid	444412	HMDB0001396	460.193	8.95	-3.1E-01	-4.2E-01	6.5E-01	-2.4E-01	3.8E-01	1.1E-01	1.8E-01	8.5E-02	-2.3E-01

MT, migration time;PC, principal component;RT, retention time
