# Supplementary material for: Amelioration of Insulin Resistance by Whey Protein in a High-Fat Diet-Induced Pediatric Obesity Male Mouse Model
Source: Nutrients. 2024 May 25;16(11):1622. doi: 10.3390/nu16111622 (PMC11174045; doi:10.3390/nu16111622)
Supplement: Supplementary file 1 [file nutrients-16-01622-s001.zip › Supplementary Tables S3.pdf]

Supplementary Table S3. Results of comparative analysis

ID	Metabolite	PubChem CID	HMDB ID	Concentration (nmol/g)														Comparative Analysis		
				control					treatment					control		treatment		treatment vs control		
				Casein1	Casein2	Casein3	Casein7	Casein8	Whey1	Whey3	Whey4	Whey5	Whey7	Mean	S.D.	Mean	S.D.	Ratio ¶	*p*-value ‖	
A_0006	2-Hydroxybutyric acid	440864	HMDB000	28	23	37	28	18	42	26	30	35	25	27	6.8	32	7.0	1.2	0.29	
A_0025	2-Oxoglutaric acid	51	HMDB000	19	23	N.D.	N.D.	N.D.	33	27	28	24	36	21	2.8	29	4.5	1.4	0.05	
A_0009	2-Oxoisovaleric acid	49	HMDB000	N.D.	N.D.	N.D.	N.D.	N.D.	N.D.	N.D.	N.D.	N.D.	N.D.	N.A.	N.A.	N.A.	N.A.	N.A.	N.A.	
A_0053	2-Phosphoglyceric acid	439278	HMDB000	N.D.	N.D.	N.D.	N.D.	N.D.	N.D.	N.D.	N.D.	N.D.	N.D.	N.A.	N.A.	N.A.	N.A.	N.A.	N.A.	
A_0005	3-Hydroxybutyric acid	441	HMDB000	254	259	407	295	338	327	470	609	260	250	310	64	383	154	1.2	0.37	
A_0052	3-Phosphoglyceric acid	439183	HMDB000	14	18	17	13	26	32	23	31	22	28	18	5.1	27	4.3	1.5	0.01	*
A_0082	6-Phosphogluconic acid	91493	HMDB000	73	55	42	40	51	69	33	54	48	57	52	13	52	13	1.0	0.98	
A_0109	Acetyl CoA_divalent	444493		N.D.	N.D.	N.D.	N.D.	18	N.D.	N.D.	N.D.	N.D.	N.D.	18	N.A.	N.A.	N.A.	<1	N.A.	
C_0059	Adenine	190	HMDB000	10	13	11	12	5.5	15	5.8	18	8.7	7.0	10	2.9	11	5.2	1.0	0.87	
C_0134	Adenosine	60961	HMDB000	59	172	139	74	83	182	161	138	273	162	105	48	183	53	1.7	0.04	*
A_0115	ADP	6022	HMDB000	369	518	550	374	731	437	441	379	544	563	508	149	473	78	0.9	0.66	
C_0010	Ala	602	HMDB000	2,316	3,688	2,931	3,364	2,647	3,247	1,263	2,874	2,567	2,509	2,989	549	2,492	747	0.8	0.27	
A_0099	AMP	6083	HMDB000	3,131	4,037	3,593	3,385	4,197	2,906	3,584	2,058	4,236	2,903	3,669	445	3,137	818	0.9	0.25	
C_0062	Anthranilic acid	227	HMDB000	N.D.	N.D.	N.D.	N.D.	N.D.	N.D.	N.D.	N.D.	N.D.	N.D.	N.A.	N.A.	N.A.	N.A.	N.A.	N.A.	
C_0092	Arg	6322	HMDB000	2.3	N.D.	N.D.	N.D.	N.D.	N.D.	2.4	N.D.	N.D.	3.2	2.3	N.A.	2.8	0.6	1.2	N.A.	
C_0055	Asn	236	HMDB000	179	139	115	97	94	160	103	154	147	140	125	35	141	23	1.1	0.43	
C_0058	Asp	424	HMDB000	303	673	1,177	569	631	647	462	548	919	683	671	318	652	173	1.0	0.91	
A_0124	ATP	5957	HMDB000	61	94	131	52	185	109	84	103	101	159	105	55	111	29	1.1	0.83	
C_0033	Betaine	247	HMDB000	651	677	966	1,013	883	872	380	732	915	413	838	166	662	252	0.8	0.23	
C_0036	Betaine aldehyde_+H_2O	249		13	7.5	8.8	5.6	3.4	8.5	4.5	10	8.1	3.8	7.6	3.5	7.0	2.7	0.9	0.78	
A_0095	cAMP	6076	HMDB000	N.D.	N.D.	N.D.	N.D.	N.D.	N.D.	N.D.	N.D.	N.D.	N.D.	N.A.	N.A.	N.A.	N.A.	N.A.	N.A.	
C_0121	Carnosine	439224	HMDB000	N.D.	N.D.	N.D.	N.D.	N.D.	N.D.	N.D.	N.D.	N.D.	N.D.	N.A.	N.A.	N.A.	N.A.	N.A.	N.A.	
A_0107	CDP	6132	HMDB000	N.D.	N.D.	N.D.	N.D.	N.D.	N.D.	N.D.	N.D.	N.D.	N.D.	N.A.	N.A.	N.A.	N.A.	N.A.	N.A.	
A_0098	cGMP	24316	HMDB000	N.D.	N.D.	N.D.	N.D.	N.D.	N.D.	N.D.	N.D.	N.D.	N.D.	N.A.	N.A.	N.A.	N.A.	N.A.	N.A.	
C_0022	Choline	305	HMDB000	525	652	951	913	375	731	390	1,019	619	405	683	248	633	260	0.9	0.76	
A_0046	*cis*-Aconitic acid	643757	HMDB000	7.0	8.2	N.D.	3.8	7.5	N.D.	N.D.	8.9	N.D.	7.9	6.6	1.9	8.4	0.7	1.3	0.19	
A_0059	Citric acid	311	HMDB000	181	244	115	113	249	135	77	204	111	209	180	66	147	58	0.8	0.42	
C_0094	Citrulline	9750	HMDB000	22	47	47	31	44	34	47	61	36	53	38	11	46	11	1.2	0.30	
A_0091	CMP	6131	HMDB000	65	70	49	48	89	57	70	75	80	53	64	17	67	12	1.0	0.77	
A_0103	CoA_divalent	87642		145	194	187	221	249	207	163	178	201	162	199	39	182	21	0.9	0.42	
C_0051	Creatine	586	HMDB000	153	235	213	246	183	236	220	224	132	434	206	38	249	111	1.2	0.45	
C_0029	Creatinine	588	HMDB000	21	11	5.1	4.8	9.9	7.3	4.2	13	5.8	4.4	10	6.6	6.9	3.5	0.7	0.33	
A_0120	CTP	6176	HMDB000	N.D.	N.D.	N.D.	N.D.	N.D.	N.D.	N.D.	N.D.	N.D.	N.D.	N.A.	N.A.	N.A.	N.A.	N.A.	N.A.	
C_0038	Cys	594	HMDB000	7.9	13	8.7	7.2	6.4	12	11	8.2	6.6	13	8.6	2.5	10	2.6	1.2	0.41	
C_0124	Cytidine	6175	HMDB000	27	27	26	7.3	7.3	31	20	55	33	23	19	11	33	14	1.7	0.13	
C_0026	Cytosine	597	HMDB000	N.D.	N.D.	N.D.	N.D.	N.D.	N.D.	N.D.	N.D.	N.D.	N.D.	N.A.	N.A.	N.A.	N.A.	N.A.	N.A.	
A_0123	dATP	15993	HMDB000	N.D.	N.D.	N.D.	N.D.	N.D.	N.D.	N.D.	N.D.	N.D.	N.D.	N.A.	N.A.	N.A.	N.A.	N.A.	N.A.	
A_0118	dCTP	65091	HMDB000	N.D.	N.D.	N.D.	N.D.	N.D.	N.D.	N.D.	N.D.	N.D.	N.D.	N.A.	N.A.	N.A.	N.A.	N.A.	N.A.	
A_0040	Dihydroxyacetone phosphate	668	HMDB000	398	25	16	15	30	24	N.D.	41	9.8	21	97	169	24	13	0.2	0.39	
A_0106	dTDP	164628	HMDB000	N.D.	N.D.	N.D.	N.D.	N.D.	N.D.	N.D.	N.D.	N.D.	N.D.	N.A.	N.A.	N.A.	N.A.	N.A.	N.A.	
A_0090	dTMP	9700	HMDB000	N.D.	N.D.	N.D.	N.D.	N.D.	N.D.	N.D.	N.D.	N.D.	N.D.	N.A.	N.A.	N.A.	N.A.	N.A.	N.A.	
A_0119	dTTP	64968	HMDB000	N.D.	N.D.	N.D.	N.D.	N.D.	N.D.	N.D.	N.D.	N.D.	N.D.	N.A.	N.A.	N.A.	N.A.	N.A.	N.A.	
A_0065	Erythrose 4-phosphate	122357	HMDB000	N.D.	N.D.	N.D.	N.D.	N.D.	N.D.	N.D.	N.D.	N.D.	N.D.	N.A.	N.A.	N.A.	N.A.	N.A.	N.A.	
A_0097	Fructose 1,6-diphosphate	172313	HMDB000	128	N.D.	N.D.	N.D.	N.D.	N.D.	N.D.	N.D.	N.D.	N.D.	128	N.A.	N.A.	N.A.	<1	N.A.	
A_0079	Fructose 6-phosphate	603	HMDB000	58	15	10	8.3	33	17	6.6	13	11	23	25	21	14	6.2	0.6	0.32	
A_0008	Fumaric acid	444972	HMDB000	498	422	209	142	190	328	179	348	313	273	292	157	288	67	1.0	0.96	
C_0019	GABA	119	HMDB000	24	13	11	6.9	14	13	9.3	9.0	11	11	14	6.5	11	1.6	0.8	0.35	
A_0116	GDP	8977	HMDB000	22	25	33	22	28	20	19	26	30	33	26	4.5	26	6.1	1.0	0.90	
C_0072	Gln	738	HMDB000	3,463	2,725	2,625	2,471	4,073	4,796	2,463	4,800	3,478	4,609	3,071	677	4,029	1,034	1.3	0.13	
C_0075	Glu	611	HMDB000	1,288	1,411	2,801	1,395	1,767	1,965	1,037	1,764	2,110	1,948	1,732	624	1,765	425	1.0	0.93	
A_0064	Gluconic acid	10690	HMDB000	1,827	2,078	2,531	1,766	1,419	1,385	1,604	1,866	2,069	1,661	1,924	413	1,717	261	0.9	0.38	
A_0076	Glucose 1-phosphate	65533	HMDB000	73	20	11	9.6	33	20	9.8	24	14	20	29	26	17	5.6	0.6	0.38	
A_0077	Glucose 6-phosphate	5958	HMDB000	200	40	25	31	228	42	15	38	35	61	105	100	38	16	0.4	0.21	
C_0144	Glutathione (GSH)	124886	HMDB000	6,587	7,382	5,553	5,275	4,762	6,964	3,867	4,656	5,743	4,700	5,912	1,057	5,186	1,197	0.9	0.34	
C_0143	Glutathione (GSSG)_divalent	65359		923	920	993	1,036	1,186	864	556	1,049	941	823	1,012	109	847	184	0.8	0.13	
C_0005	Gly	750	HMDB000	2,060	2,126	2,174	1,726	1,949	2,786	1,950	2,623	2,845	2,419	2,007	178	2,525	361	1.3	0.03	*
A_0039	Glyceraldehyde 3-phosphate	729	HMDB000	N.D.	N.D.	N.D.	N.D.	N.D.	N.D.	N.D.	N.D.	N.D.	N.D.	N.A.	N.A.	N.A.	N.A.	N.A.	N.A.	
A_0041	Glycerol 3-phosphate	439162	HMDB000	1,505	1,798	3,522	2,487	3,833	2,572	2,085	1,854	2,244	2,660	2,629	1,027	2,283	336	0.9	0.51	
A_0002	Glycolic acid	757	HMDB000	N.D.	N.D.	N.D.	N.D.	N.D.	N.D.	N.D.	N.D.	N.D.	N.D.	N.A.	N.A.	N.A.	N.A.	N.A.	N.A.	
A_0001	Glyoxylic acid	760	HMDB000	N.D.	N.D.	N.D.	N.D.	N.D.	N.D.	N.D.	N.D.	N.D.	N.D.	N.A.	N.A.	N.A.	N.A.	N.A.	N.A.	
A_0101	GMP	6804	HMDB000	454	499	508	445	587	441	454	381	583	467	498	57	465	74	0.9	0.45	
A_0126	GTP	6830	HMDB000	6.7	6.5	14	N.D.	12	N.D.	6.6	11	10	16	9.9	3.8	11	3.7	1.1	0.74	
C_0079	Guanine	764	HMDB000	N.D.	N.D.	N.D.	N.D.	N.D.	N.D.	1.0	N.D.	N.D.	N.D.	N.A.	N.A.	1.0	N.A.	1<	N.A.	
C_0139	Guanosine	6802	HMDB000	18	33	26	13	15	34	20	47	48	29	21	8.3	36	12	1.7	0.06	
C_0082	His	773	HMDB000	618	782	660	716	641	852	523	776	782	628	683	66	712	134	1.0	0.68	
C_0034	Homoserine	12647	HMDB000	2.9	N.D.	5.9	N.D.	5.5	3.6	N.D.	5.1	4.7	N.D.	4.8	1.6	4.5	0.7	0.9	0.79	
C_0049	Hydroxyproline	5810	HMDB000	32	44	48	35	34	34	29	35	43	34	39	6.8	35	5.1	0.9	0.36	
C_0060	Hypoxanthine	790	HMDB000	311	358	202	197	79	302	170	627	221	131	229	109	290	199	1.3	0.57	
C_0052	Ile	791	HMDB000	253	289	245	233	134	334	188	310	229	209	231	58	254	64	1.1	0.56	
A_0100	IMP	8582	HMDB000	584	231	99	293	558	340	188	166	185	381	353	211	252	101	0.7	0.37	
C_0135	Inosine	6021	HMDB000	864	1,002	493	649	266	1,038	471	1,836	733	477	655	292	911	567	1.4	0.40	
A_0058	Isocitric acid	1198	HMDB000	N.D.	N.D.	N.D.	N.D.	N.D.	N.D.	N.D.	N.D.	N.D.	N.D.	N.A.	N.A.	N.A.	N.A.	N.A.	N.A.	
A_0004	Lactic acid	612	HMDB000	17,176	15,669	9,808	12,137	12,466	14,179	6,132	10,085	8,741	14,164	13,451	2,948	10,660	3,506	0.8	0.21	
C_0053	Leu	857	HMDB000	513	526	450	405	268	600	371	575	465	401	433	104	482	102	1.1	0.47	
C_0073	Lys	866	HMDB000	838	1,151	1,023	806	623	1,199	854	1,098	1,222	821	888	204	1,039	190	1.2	0.26	
A_0020	Malic acid	525	HMDB000	1,525	1,196	607	297	414	997	502	1,092	851	812	808	529	851	225	1.1	0.87	
A_0114	Malonyl CoA_divalent	644066		N.D.	N.D.	N.D.	N.D.	N.D.	N.D.	N.D.	N.D.	N.D.	N.D.	N.A.	N.A.	N.A.	N.A.	N.A.	N.A.	
C_0077	Met	876	HMDB000	102	126	99	66	52	120	92	145	135	85	89	30	115	26	1.3	0.18	
C_0020	*N*,*N*-Dimethylglycine	673	HMDB000	32	36	25	40	39	51	22	37	30	37	35	6.0	36	11	1.0	0.87	
A_0134	NAD^+	5893	HMDB000	46	123	555	304	442	237	217	104	260	371	294	213	238	96	0.8	0.61	
A_0137	$NADP^+$	5886	HMDB000	22	54	102	78	127	57	52	28	87	79	76	41	60	23	0.8	0.48	
C_0056	Ornithine	389	HMDB000	569	507	783	499	357	468	436	587	477	442	543	155	482	61	0.9	0.45	
C_0088	Phe	994	HMDB000	184	177	139	134	81	200	128	199	141	137	143	41	161	35	1.1	0.47	
A_0037	Phosphoenolpyruvic acid	1005	HMDB000	N.D.	N.D.	N.D.	N.D.	N.D.	N.D.	N.D.	N.D.	N.D.	N.D.	N.A.	N.A.	N.A.	N.A.	N.A.	N.A.	
C_0030	Pro	614	HMDB000	254	318	259	242	173	326	188	321	247	264	249	52	269	57	1.1	0.58	
A_0104	PRPP	7339	HMDB000	N.D.	N.D.	N.D.	N.D.	N.D.	N.D.	N.D.	N.D.	N.D.	N.D.	N.A.	N.A.	N.A.	N.A.	N.A.	N.A.	
C_0008	Putrescine	1045	HMDB000	13	14	4.8	10	6.5	6.1	4.1	5.2	5.1	3.5	9.6	3.9	4.8	1.0	0.5	0.05	*
A_0003	Pyruvic acid	1060	HMDB000	231	363	280	308	306	337	255	201	331	328	298	48	291	60	1.0	0.84	
A_0072	Ribose 5-phosphate	439167	HMDB000	70	75	35	41	74	51	54	40	69	47	59	19	52	11	0.9	0.52	
A_0073	Ribulose 5-phosphate	439184	HMDB000	484	573	304	470	280	631	395	764	464	401	422	126	531	161	1.3	0.27	
C_0152	*S*-Adenosylmethionine	34755	HMDB000	75	60	37	58	57	51	33	33	47	33	57	14	39	9.0	0.7	0.04	*
C_0009	Sarcosine	1088	HMDB000	27	25	16	31	22	48	20	60	23	53	24	5.5	41	18	1.7	0.12	
A_0084	Sedoheptulose 7-phosphate	165007	HMDB000	93	116	55	77	68	99	84	138	83	81	82	24	97	24	1.2	0.34	
C_0023	Ser	617	HMDB000	391	374	488	310	390	440	273	342	546	331	391	64	386	107	1.0	0.94	
C_0071	Spermidine	1102	HMDB000	25	19	13	28	24	22	15	31	22	12	22	5.9	20	7.5	0.9	0.74	
C_0112	Spermine	1103	HMDB000	7.0	5.6	3.7	6.5	6.7	5.6	4.4	6.7	8.4	3.0	5.9	1.3	5.6	2.1	0.9	0.79	
A_0011	Succinic acid	1110	HMDB000	468	1,144	1,628	1,546	1,214	1,257	454	794	904	1,292	1,200	459	940	348	0.8	0.35	
C_0035	Thr	6288	HMDB000	386	404	379	279	230	472	245	394	445	332	336	77	378	91	1.1	0.45	
C_0123	Thymidine	5789	HMDB000	N.D.	N.D.	N.D.	N.D.	N.D.	N.D.	N.D.	N.D.	N.D.	N.D.	N.A.	N.A.	N.A.	N.A.	N.A.	N.A.	
C_0045	Thymine	1135	HMDB000	N.D.	N.D.	N.D.	N.D.	N.D.	N.D.	N.D.	N.D.	N.D.	N.D.	N.A.	N.A.	N.A.	N.A.	N.A.	N.A.	
C_0115	Trp	1148	HMDB000	47	58	48	44	35	65	43	57	48	55	46	8.4	54	8.4	1.2	0.21	
C_0100	Tyr	1153	HMDB000	220	246	160	168	114	229	154	214	211	171	182	52	196	32	1.1	0.62	
C_0064	Tyramine	5610	HMDB000	N.D.	N.D.	N.D.	N.D.	N.D.	N.D.	N.D.	N.D.	N.D.	N.D.	N.A.	N.A.	N.A.	N.A.	N.A.	N.A.	
A_0108	UDP	6031	HMDB000	13	20	27	12	24	17	13	20	27	26	19	6.8	20	6.0	1.1	0.80	
A_0093	UMP	6030	HMDB000	500	468	491	461	707	341	471	361	784	464	525	103	484	178	0.9	0.67	
C_0028	Uracil	1174	HMDB000	41	40	26	25	9.8	35	21	61	31	28	29	13	35	15	1.2	0.48	
C_0125	Uridine	6029	HMDB000	257	234	172	171	82	220	92	331	170	141	183	68	191	91	1.0	0.89	
A_0121	UTP	6133	HMDB000	N.D.	N.D.	7.8	N.D.	8.0	5.1	N.D.	4.9	5.1	10	7.9	0.12	6.2	2.5	0.8	0.28	
C_0032	Val	1182	HMDB000	494	550	495	456	284	643	380	593	490	436	456	101	508	109	1.1	0.45	
C_0011	β-Ala	239	HMDB000	208	319	395	346	116	336	191	332	341	216	277	113	283	73	1.0	0.92	

N.A., Not Available; N.D., Not Detected

¶ The ratio of the detected mean values between the two groups.

|| Welch's t-test (*p<0.001)
